# Rapid detection of bacterial infection using a novel single-tube, four-colour flow cytometric method: Comparison with PCT and CRP

**DOI:** 10.1016/j.ebiom.2021.103724

**Published:** 2021-11-26

**Authors:** Jari Nuutila, Ulla Hohenthal, Jarmo Oksi, Päivi Jalava-Karvinen

**Affiliations:** a Department of Life Technologies, University of Turku, Turku, Finland; b Turku University Hospital, Department of Medicine, Turku, Finland; c Faculty of Medicine, University of Turku, Turku, Finland.

**Keywords:** Flow cytometry, Host biomarker, Differential diagnostic, Bacterial infection, Viral infection

## Abstract

**Background:**

A key factor behind the unnecessary use of antibiotics is the lack of rapid and accurate diagnostic tests. In this study, we developed a novel and fast flow cytometric single-tube method to detect bacterial infections within 30 minutes.

**Methods:**

Quantitative flow cytometric four-colour analysis of host biomarkers CD35, CD64, CD329, and MHC class I expression on neutrophils and lymphocytes was performed on samples taken from 841 febrile patients with suspected infection. Obtained data was incorporated into the four-colour bacterial infection (FCBI)-index, using the developed bacterial infection algorithm.

**Findings:**

In distinguishing between microbiologically confirmed bacterial (n = 193) and viral (n = 291) infections, the FCBI-index method was superior to serum C-reactive protein (CRP) and procalcitonin (PCT). In 269 confirmed viral respiratory tract infections, 43% (95% CI: 37–49%) of the patients had an increased FCBI-index, suggesting probable bacterial coinfection.

**Interpretation:**

The proposed FCBI-index test might be a potent additional tool when assessing appropriateness of empiric antibiotic treatment.

**Funding:**

This study has been financially supported by Turku University Hospital (Turku, Finland) and The Finnish Medical Foundation.

**Research in context**

**Evidence before this study**

There is an ongoing need for new sensitive and specific markers of bacterial infection. One candidate is the flow cytometric determination of host biomarkers, i.e. receptors, on the surface of blood leukocytes. The hypothesis behind the idea is that bacterial and viral infections induce different systemic profiles of proinflammatory cytokines, which can lead to different expression patterns of certain cell surface receptors on blood leukocytes in these different infection types. This study is a continuation of our previous works on this novel and narrow field of research.

**Added value of this study**

As a result of persistent development work during the last two decades, we have now developed a novel and simple flow cytometry-based bacterial infection marker, the four-colour bacterial infection (FCBI)-index, which incorporates the quantitative analysis of CD35, CD64 and CD329 receptors on neutrophils and lymphocytes and detects febrile bacterial infections reliably in less than 30 minutes. Data of this comparative study clearly shows that the FCBI-index method is superior to CRP and PCT when distinguishing between bacterial and viral infections.

**Implications of all the available evidence**

The FCBI-index might be a potent part of antimicrobial stewardship measures in hospitals and health centers, helping physicians to decide whether empiric antibiotic treatment is necessary or not.

Alt-text: Unlabelled box

## 1 Introduction

A key factor behind the unnecessary use of antibiotics and the increasing global burden of antimicrobial resistance is the lack of rapid and accurate diagnostic tests [1]. Therefore, there is an ongoing need for the discovery of new sensitive and specific markers of bacterial infection. One candidate is the flow cytometric determination of host markers, receptors, on the surface of blood leukocytes. The hypothesis behind the idea is that peripheral blood leukocytes act as biosensors responding to pathogen-induced (e.g. a bacterium or virus) changes in the systemic cytokine profile [2] by altering the expression pattern of certain cell surface receptors.

Until now, the increased expression of Fc-gamma-receptor I (FcγRI/CD64) on neutrophils has been the most widely used (and only) commercially available flow cytometric marker of infection (both bacterial and viral), as well as severity of sepsis [3], [4], [5], [6]. Besides being a sensitive marker for the detection of bacterial infection, the increased expression of CD64 on neutrophils can also occur in virus infections (especially in DNA virus infections) and thus cannot be used unambiguously to differentiate between bacterial and viral diseases[ 7,8].

Compared with neutrophil CD64, neutrophil complement receptor 1 (CR1/CD35) seems to be a more specific bacterial infection marker. In 2006, we discovered that the average expression level of CD35 on neutrophils in bacterial infections was over three-fold higher than in viral infections and healthy controls, displaying 85% sensitivity and specificity when distinguishing between bacterial and viral infections [9]. In the same article, the neutrophil CD35-based differentiation between bacterial and viral infections was further improved by generating the Clinical Infection Score (CIS) point, incorporating four variables, the quantitative flow cytometric analysis of CD35 and CD11b (complement receptor 3, CR3) on neutrophils and standard clinical laboratory data, serum CRP level and erythrocyte sedimentation rate. CIS point displayed 98% sensitivity and 97% specificity when distinguishing between bacterial and viral infections. From this particular study, we learned that any single bacterial infection marker alone cannot be used to reliably differentiate between bacterial and viral infections and that diagnostic accuracy can be improved by a combination of several (3-4) bacterial infection markers.

Since 2006, we have developed three more (flow cytometric) multiparametric score/index methods to distinguish between bacterial and viral infections: Bacterial Infection Score (BIS), which was the first pure flow cytometric bacterial infection marker, Bacterial Infection (BI)-index, and Two-Colour Bacterial Infection (TC-BI)-index [10], [11], [12].

Albeit highly sensitive and specific, a common drawback of the above-mentioned early multiparametric methods is their complexity. For example, the receptor analyses were conducted using isolated leukocytes, widening the time window from procuring blood samples to data handling and index/score-based diagnosis up to 45 min. In addition, one- or two-colour flow cytometric receptor analyses were performed in 2-3 separate test tubes. The lack of the universal leukocyte marker was still one more problem in these early score/index methods.

Based on what we have learned from our previous works in this field, we can conclude that there are three basic principles underlying the concept of an efficient flow cytometric bacterial infection marker: It should be 1) multiparametric, 2) fast and practical with the minimum amount of sample handling, and 3) require only one measurement from a single test tube. Consequently, a novel state-of-the-art bacterial infection marker fulfilling all of the above criteria has now been developed. We present in this study the truly workable multiparametric flow cytometry method, the Four-Colour Bacterial Infection (FCBI)-index, which is based on detection of the relative number of CD35, CD64 and CD329 receptors on neutrophils and lymphocytes.

In order to reduce the total sample handling time from over 45 min (above-mentioned previous methods) to less than 30 min in the present study, the incubation of the whole blood sample (instead of isolated leukocytes) with four fluorescent labelled receptor specific monoclonal antibodies, red blood cell lysis, and the flow cytometric run were performed consecutively in the same test tube without any time-consuming washing steps. In the data handling process, the conversion of the raw receptor expression data into a single FCBI-index value was performed using the novel bacterial infection algorithm.

Of the studied inflammatory receptors, CD35 is expressed on granulocytes, monocytes, B cells, follicular dendritic cells, erythrocytes, NK cells and at low levels on T cells [13]. It is a phagocytosis receptor that also disrupts the autologous complement activation process at the site of infection [14], [15], [16].

CD64 is expressed on monocytes, macrophages, circulating dendritic cells, and at low levels on resting neutrophils and lymphocytes [17]. One of its main functions is to modulate the kinetics of both receptor-mediated endocytosis and phagocytosis [18].

Sialic acid-binding Ig-like lectin 9 (Siglec9/CD329) is a member of the sialic acid-recognition protein family that is highly expressed on neutrophils and monocytes, and only weakly on lymphocytes and NK cells [19]. CD329 is involved in the pathogenesis of sepsis through its interaction with Toll-like receptor 4, regulating the polarization of macrophages and inhibiting the stimulation of neutrophils [20]. It has also been reported to take part in leukocyte migration from the blood into the sites of inflammation, through binding to vascular adhesion protein-1 (VAP-1/AOC3) on vascular endothelial cells [21].

Major histocompatibility complex I (MHCI) molecules, which bind endogenous (viral) antigen peptides and present them to the CD8-positive cytotoxic T-cells [22,23], are found on the cell surface of all nucleated cells in the bodies of vertebrates. The role of the fluorescent-labelled anti-MHCI antibody in the presented method is to separate fluorescent neutrophil and lymphocyte populations from non-fluorescent debris.

In this comparative study, we present the distribution of FCBI-index, CRP and PCT values primarily in bacteremia, microbiologically confirmed local bacterial infection, clinically diagnosed probable bacterial infection, and microbiologically confirmed viral respiratory tract infection (RTI).

## 2 Methods

### 2.1 Ethics and recruitment of study subjects

The study protocol was approved by the Ethical Committee of Turku University Hospital district (Reference number: ETMK 23.11.2007 § 99). All research was performed in accordance with relevant guidelines and regulations. The study period covered four winter influenza seasons (October to March) from 2015 to 2019.

Adult patients (aged 18 years or over) with a suspected infection were eligible for the study if they had a body temperature of at least 37.5°C. Patients with ongoing cytostatic treatment for a malignancy or biological treatment against autoimmune diseases such as rheumatoid arthritis or an inflammatory bowel disease were excluded, as well as patients with hematological malignancies with possible influence on white blood cells, and those who were hospitalized for over 48 h before procuring blood samples.

Informed consent was obtained from all participants after the nature and possible consequences of the study were explained, both in writing and orally. The patient volunteers, as well as healthy controls, signed an approval form to give a further 3 mL lithium heparin blood sample for the receptor study. The sample was taken at the same time as the routine tests.

The clinical patient data were collected from the confidential patient records by authorized clinician. The patients had the option to withdraw at any time during the study.

The patients were treated according to normal clinical policy and the receptor analysis (FCBI-index value) had no influence on the treatment decisions.

### 2.2 Diagnoses

For the purpose of this study, we attempted to confirm all diagnoses either microbiologically or serologically. It should be noted that the used methods were chosen on a clinical basis, so we did not have the opportunity to study all pathogens systematically.

Bacterial culture served as a gold standard for the diagnosis of confirmed bacterial infections (n = 193). Confirmed bacterial infections included cases where bacterial culture from sources such as blood, urine, stool or needle aspiration from an abscess or empyema was positive. In addition to bacterial culturing methods, a positive bacterial antigen test was also accepted (such as a urine pneumococcal antigen test), if the clinical assessment supported the finding. A concomitant viral infection was diagnosed in 5.7% (11/193) of cases in this group (Supplementary Fig. 8).

Confirmed viral infections (n = 291) were diagnosed using serological tests, PCR tests, or antigen detection tests. Confirmed viral infection required detection of IgM antibodies or an at least 4-fold increase in IgG antibody titer in the serum. At the start of the study, influenza was mainly diagnosed using the antigen detection test, but later, PCR tests became the main method for diagnosis of infection with influenza virus or other respiratory viruses. A concomitant asymptomatic bacteriuria (negligible bacteria level in urine) was diagnosed in 5.2% (15/291) of cases in this group (Supplementary Fig. 8).

**Fig. 1:**
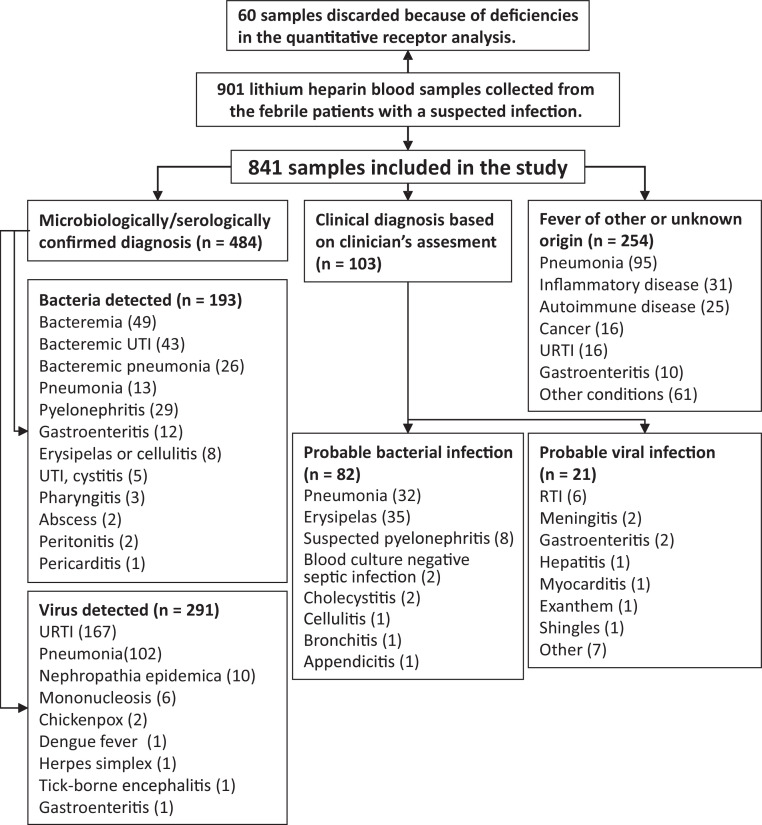
Enrollment and follow-up of 901 febrile patients suspected with infection. Details regarding disease-specific pathogens and subgroup-specific antimicrobial prescriptions are provided in the Supplementary Tables 1, 2 and 3. URTI: upper respiratory tract infection, RTI: respiratory tract infection, UTI: urinary tract infection.

In addition, we also accepted some clinical diagnoses without definitive microbiological test results. Clinically diagnosed probable bacterial (n = 82) or viral (n = 21) infections were classified by an infectious disease specialist and were mainly cases of pneumonia and erysipelas infections of skin (Fig. 1). Patients whose chest radiograph showed lobar pneumonia or had pneumonia with CRP higher than 200 mg/L were classified as probable bacterial pneumonias (n = 32). In total, 16 of 102 patients (16%) who were diagnosed with confirmed viral pneumonia also fulfilled the criteria for probable bacterial pneumonia. Clinically diagnosed probable viral infections included shingles and chicken pox (Varicella-zoster virus infections).

The second biggest group of the study was composed of the patients with fever of other or unknown origin (n = 254). This heterogeneous group included patients with various diseases, such as pneumonia with unknown etiology (not fulfilling the criteria of probable bacterial pneumonia described above), inflammatory diseases and cancer (Fig. 1).

At least two clinicians, one of which was an infectious disease specialist, made the final diagnosis.

Note that the FCBI-index test was not used for making a clinical diagnosis.

Disease-specific bacterial and viral pathogens are listed in Supplementary Tables 1 and 2, respectively.

Group-specific antimicrobial prescriptions are listed in Supplementary Table 3.

### 2.3 Routine laboratory tests

Demographic and clinical characteristics of the patients at baseline are provided in Supplementary Table 4. Routine laboratory tests (PCT, CRP and leukocyte count) were performed at the clinical laboratory (TYKSlab) of the Turku University Central Hospital. Total and differential counts (× 10^9^/L) of leukocytes were determined using a Sysmex XE-2100 automatic blood cell analyzer (Sysmex, Kobe, Japan). Measurement of the serum CRP level (mg/L) was based on a whole-blood immunoturbidimetric method. An electrochemiluminescent immunoassay (ECLIA) method was used to determine serum procalcitonin (PCT) level (ng/L). All tests, including flow cytometric receptor analysis, were performed using blood samples taken at the same time.

### 2.4 CRP and PCT cutoff values used in this study

We used a CRP cutoff value of 77 mg/L, which was found to give the optimal ROC curve statistics to differentiate between microbiologically confirmed bacterial and viral infections in this and previous studies [9,10].

Based on studies by Falsey et al. and Van Nieuwkoop et al., as well as on what we learned from the PCT data in this study, a PCT cutoff value of ≥ 250 ng/L was used when detecting potential bacterial infection [24,25].

### 2.5 Description of quantitative flow cytometry-based FCBI-index method

Flow cytometry (FCM): Two CyFlow Cube 8 (Sysmex Europe GmbH, Germany) flow cytometers with slightly different measuring capacities were used for the quantitative receptor analysis of blood leukocytes. One instrument (FCM1), located in the Department of Life Technologies, University of Turku, was used to analyse blood samples taken from patients treated at different medical wards of Turku University Hospital (n = 561). The other instrument (FCM2), located in the emergency room of Turku University Hospital, was used to analyse only those blood samples taken from emergency room patients (n = 280).

Fluorescence-conjugated receptor-specific monoclonal antibodies (test kit): Phycoerythrin (PE)-conjugated anti-human CD35 (Clone E11) was purchased from Ancell (ANC-184-050), PerCP-Vio700-conjugated anti-human CD64 (Clone 10.1.1) was purchased from Miltenyi Biotec (130-101-422), fluorescein isothiocyanate (FITC)-conjugated anti-human CD329 (Clone 191240) was purchased from R&D systems (FAB1139F) and AF647-conjugated anti-human MHC class I molecule (Clone W6/32) was purchased from R&D systems (FAB7098R). (Supplementary Table 5).

**Fig. 2:**
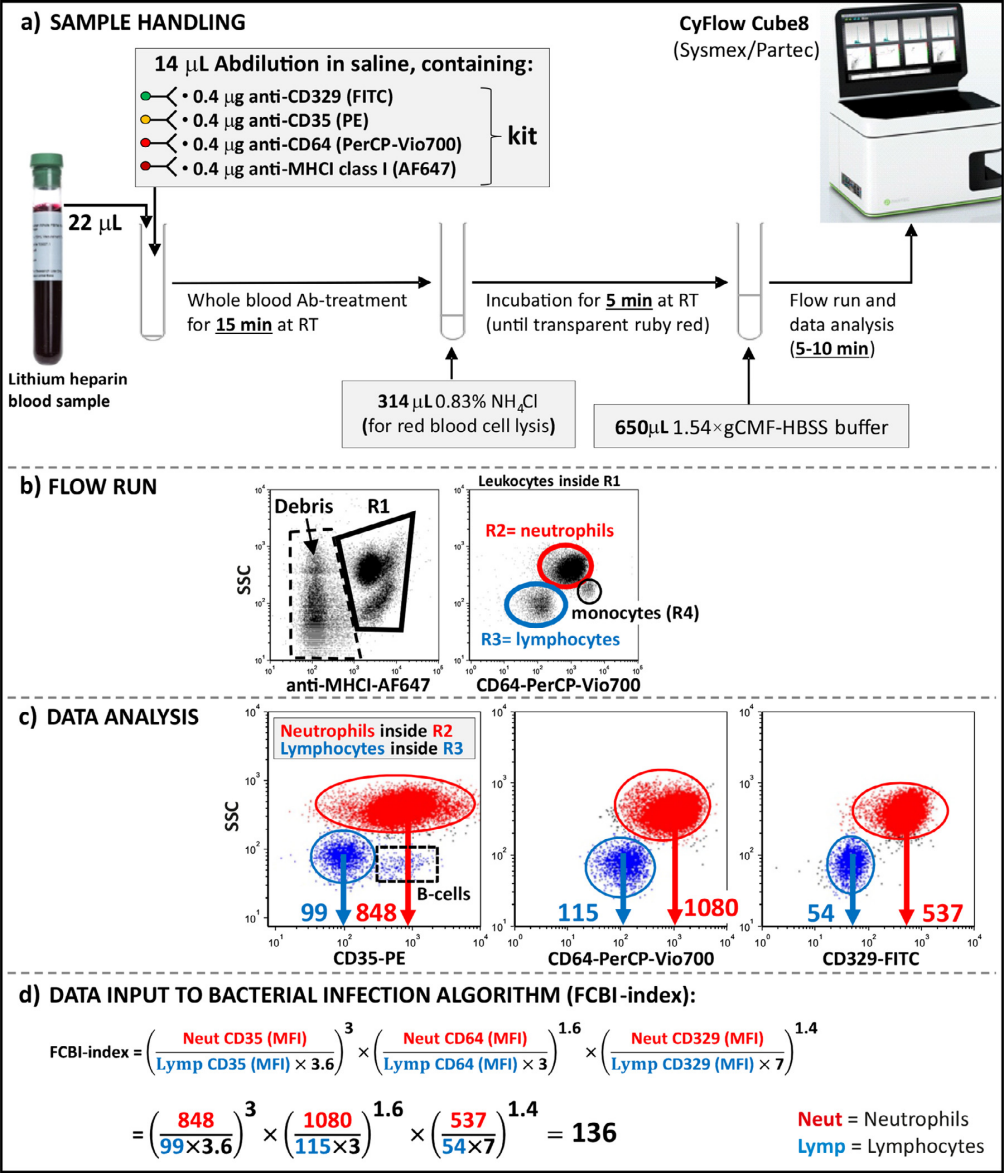
A schematic diagram on how to calculate the FCBI-index value of the patient with microbiologically confirmed *Streptococcus pneumoniae* pneumonia using the novel FCBI-index method. (a) The incubation of the whole blood sample with the receptor-specific antibodies, red blood cell lysis, and flow cytometry run were carried out one after another in the same test tube. (b) During the flow cytometry run, the leukocyte population within gate R1 was first gated out from the side scatter (SSC)/MHCI bivariate histogram, after which gates R2 (red) and R3 (blue) were set around neutrophil and lymphocyte populations, respectively, in the SSC/CD64 bivariate histogram containing leukocytes inside gate R1. The stop count of 1000 monocytes inside the monocyte gate (black/R4) was used, leading to the collection of a total of 5000–20000 leukocytes. (c) For the purpose of data analysis, three additional bivariate histograms, namely SSC/CD35, SSC/CD64, and SSC/CD329 were made, showing mean fluorescence intensities (MFI, which correlate with the number of receptors on the cell surface) of gated neutrophil (red) and lymphocyte (blue) populations. In the SSC/CD35 histogram, B-lymphocytes expressing CD35 were left outside the lymphocyte gate. (d) The actual FCBI-index value was calculated by substituting the obtained receptor-specific MFI data from neutrophil and monocyte populations into the bacterial infection algorithm. A more detailed step-by-step description of the development of the FCBI-index method is presented in the method section and Supplementary Figs. 1–5. 1.54 × gCMF-HBSS: 1.54-fold calcium- and magnesium-free HBSS -buffer supplemented with 1.54 mg/mL gelatin.

Preparation of patient blood sample for flow cytometric analysis: For quantitative receptor analysis, 22 μL of the lithium heparin anticoagulated whole blood and 14 μL of antibody dilution, containing 0.4 μg of each antibody (anti-human CD35, CD64, CD329, and MHC class I) in saline, were mixed in one polystyrene flow cytometer vial and incubated for 15 min at room temperature. Red blood cell lysis was performed by adding 314 μL of 0.83% NH_4_Cl to the blood-antibody suspension and incubating for 5 min at room temperature, after which the run volume was adjusted to 1 mL by adding 650 μL of cold 1.54-fold calcium- and magnesium-free HBSS -buffer supplemented with 1.54 mg/mL gelatin (1.54 × gCMF-HBSS) (Fig. 2a).

Gate settings in flow cytometric run: In the first phase of the flow cytometry run, the fluorescent leukocyte population within gate R1 was gated out from the SSC log/anti-MHCI log bivariate histogram (unfluorescent debris was excluded) (Fig. 2b). In the second phase of the run, additional gates R2 and R3 were applied around neutrophil and lymphocyte populations, respectively, in the SSC log/CD64 log bivariate histogram, containing leukocytes inside gate R1 (Fig. 2b). The stop count of 1000 monocytes inside the monocyte gate (R4) in the SSC/CD64 bivariate histogram was used, leading to the collection of a total of 5000–20000 leukocytes.

Flow cytometric data analysis: For the data analysis, three additional bivariate histograms, namely SSC/CD35, SSC/CD64, and SSC/CD329 were made, showing mean fluorescence intensities (MFI, which correlate with the number of receptors on the cell surface) of gated neutrophil (R2) and lymphocyte (R3) populations (Fig. 2c). In the SSC/CD35 histogram, B-lymphocytes expressing CD35 were left outside the lymphocyte gate.

Calculation of FCBI-index value: The FCBI-index value was simply calculated by substituting the obtained receptor-specific MFI data from neutrophil and lymphocyte populations into the bacterial infection algorithm (Fig. 2d):

$$\text{FCBI}-\text{index}\;={\left(\frac{\text{Neutrophil}\;\text{CD}35\;(\text{MFI})}{\text{Lymphocyte}\;\text{CD}35\;(\text{MFI})\;\times \;3.6}\right)}^{3}\times {\left(\frac{\text{Neutrophil}\;\text{CD}64\;(\text{MFI})}{\text{Lymphocyte}\;\text{CD}64\;(\text{MFI})\times 3}\right)}^{1.6}\times {\left(\frac{\text{Neutrophil}\;\text{CD}329\;(\text{MFI})}{\text{Lymphocyte}\;\text{CD}329\;(\text{MFI})\times 7}\right)}^{1.4}$$

Constant denominator (3.6, 3 and 7; receptor specific cutoff values for RATIOs) and power (3, 1.6 and 1.4) values in the algorithm were obtained by iteration (trial and error method) in order to achieve the best differentiation between bacterial and viral infections by the final FCBI-index (Supplementary Fig. 5). In order to achieve the wide dynamic range of eight decades of the method, all three variables must be incorporated into the FCBI-index. Neutrophil CD35 is the cornerstone variable of the FCBI-index, whose differential capacity is supported by the other two variables, CD64 and CD329. A more detailed step-by-step description of the development of the bacterial infection algorithm is presented in the Supplementary Figs. 1-5.

### 2.6 Reproducibility testing of the FCBI-index method

Reproducibility of the FCBI-index method was tested in two different conditions. First, we tested on how gate settings during flow cytometric data analysis (Figs. 2b and 2c) influenced on final FCBI-index values. In order to do that, we performed 10 consecutive FCBI-index calculations starting every time with fresh gate settings using the flow cytometric raw data as a test material. After performing above-mentioned calculations with six different patient data, we observed that the proportion of SD from mean value (SD%) stayed a constant low level (2-4%) regardless of mean FCBI-index value (Supplementary Table 6). Second, we tested the influence of sample handling (Incubation of the whole blood sample with the receptor-specific antibodies, red blood cell lysis, and flow cytometry run) on the reproducibility of the method by repeating the sample handling protocol simultaneously in five separate test tubes. The test was conducted using the blood of one patient sample and FCBI-index values of five separate measurements were determined using constant gate settings (Supplementary Table 7). After statistical calculations, the mean FCBI-index value of 3.41 and SD of 0.16 (SD% = 4.6) was obtained.

According to the presented evidence, it can be concluded that the reproducibility of the FCBI-index method is good.

### 2.7 Statistical analysis

**Fig. 3:**
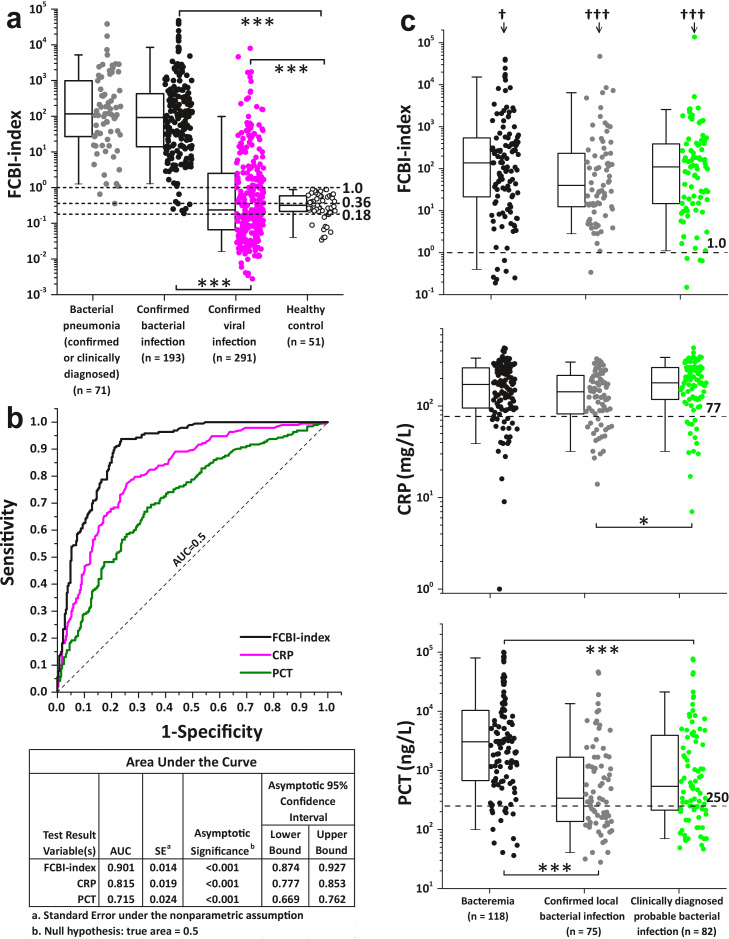
Detection of bacterial infection using the FCBI-index method. (a) The three major FCBI-index cutoff values cited in this manuscript are presented as horizontal dotted lines. The bacterial pneumonia group consisted of 71 patients with microbiologically confirmed bacteremic (n = 26) and non-bacteremic (local, n = 13) pneumonia, and clinically diagnosed probable bacterial pneumonia (n = 32) (See also Fig. 1, 6 and 7). (b) ROC curves are shown, comparing FCBI-index, CRP and PCT as methods for separating confirmed febrile bacterial infection (n = 193) from confirmed febrile viral infection (n = 291). The area under the ROC curve (AUC) statistics are contained in the table underneath Fig. 3b. (c) Distribution of the FCBI-index, CRP and PCT values in three different types of bacterial infections. The dotted horizontal lines represent the used cutoff value of the FCBI-index (1.0), CRP (77 mg/L), and PCT (250 ng/L) when detecting bacterial infection. †p < 0.05 and †††p < 0.001 denote significantly different proportions of increased parameter values (≥ cutoff values) between tested bacterial infection markers by chi-square test. (a and c) The Kolmogorov-Smirnov test showed that the presented data was not normally distributed. The Bonferroni corrected p-values of post-hoc pairwise between group comparisons were determined after the Kruskal-Wallis (one-way ANOVA on ranks) test, *p < 0.05, ***p < 0.001. All significant p-values are presented. Box plot statistics (25th and 75th percentiles, and median value) with the 5th and 95th percentile (whiskers) values are shown.

**Fig. 4:**
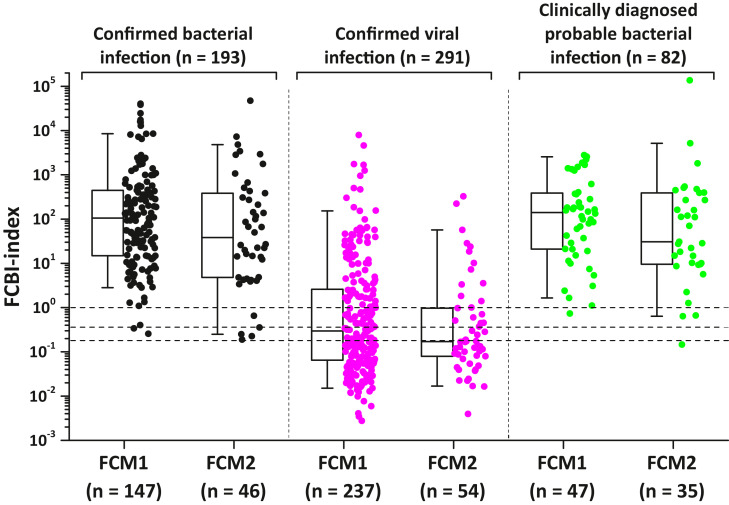
Influence of used flow cytometry (FCM1 and FCM2) on FCBI-index distribution in patients with confirmed bacterial and viral infections and clinically diagnosed probable bacterial infections. Horizontal dotted lines represent the three FCBI-index cut-off values: 0.18, 0.36 and 1.0. FCM1, located in the department of Life Technologies, University of Turku, was used to analyse blood samples taken from patients treated at different medical wards of Turku University Hospital. FCM2, located in the emergency room of Turku University Hospital, was used to analyse only those blood samples taken from emergency room patients. Box plot statistics (25th and 75th percentiles, and median value) with the 5th and 95th percentile (whiskers) values are shown.

**Fig. 5:**
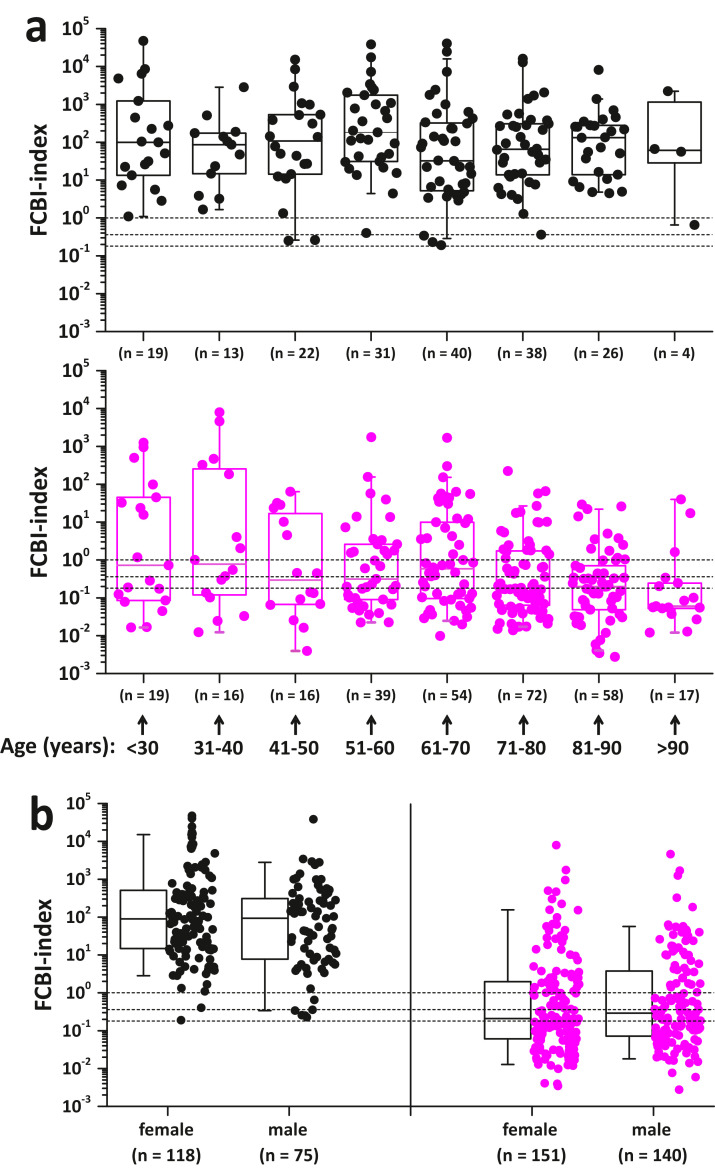
Influence of age (a) and gender (b) on FCBI-index value distribution in patients with confirmed bacterial (black symbols) and viral (magenta symbols) infections. Horizontal dotted lines represent the three FCBI-index cut-off values: 0.18, 0.36 and 1.0. Box plot statistics (25th and 75th percentiles, and median value) with the 5th and 95th percentile (whiskers) values are shown.

**Fig. 6:**
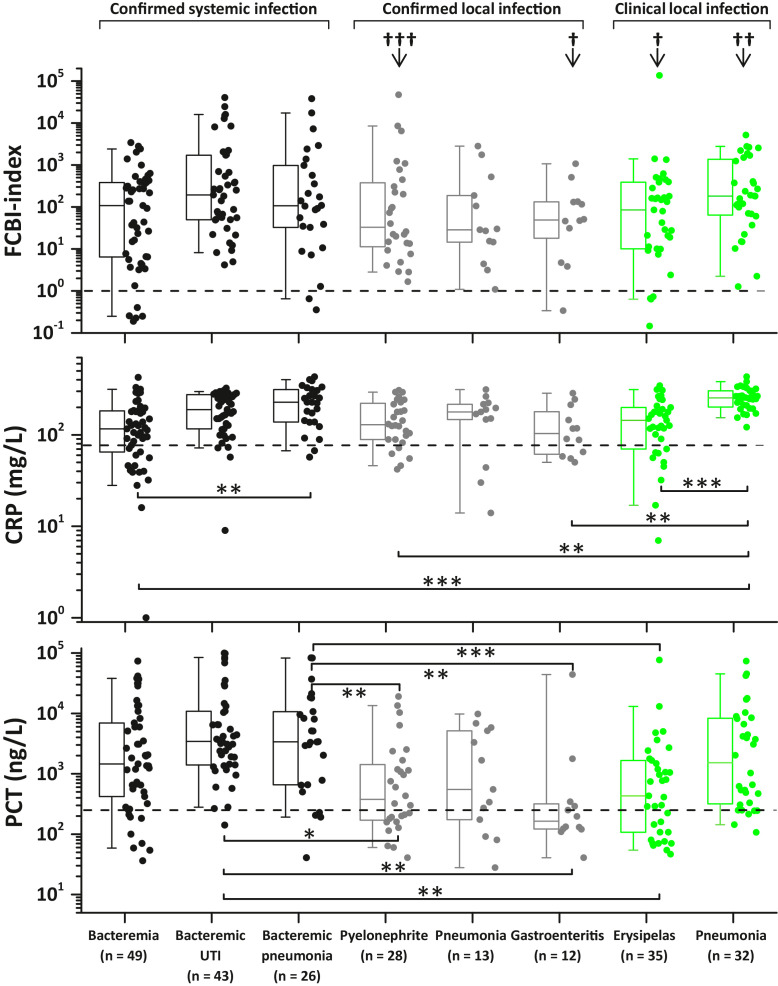
Distribution of the FCBI-index, CRP and PCT values in eight different types of bacterial infections. Only those subgroups with n > 10 are shown. The dotted horizontal line represents the optimal cutoff value of FCBI-index (1.0), CRP (77 mg/L), and PCT (250 ng/L) when detecting bacterial infection. The Kolmogorov-Smirnov test showed that the presented data was not normally distributed. The Bonferroni corrected p-values of post-hoc pairwise between group comparisons were determined after the Kruskal-Wallis (one-way ANOVA on ranks) test, *p < 0.05, **p < 0.01, ***p < 0.001. All significant p-values are presented. †p < 0.05, ††p < 0.01, and †††p < 0.001 denote significantly different proportions of increased parameter values between tested bacterial infection markers by Chi-square test. Box plot statistics (25th and 75th percentiles, and median value) with the 5th and 95th percentile (whiskers) values are shown UTI: urinary tract infection.

**Fig. 7:**
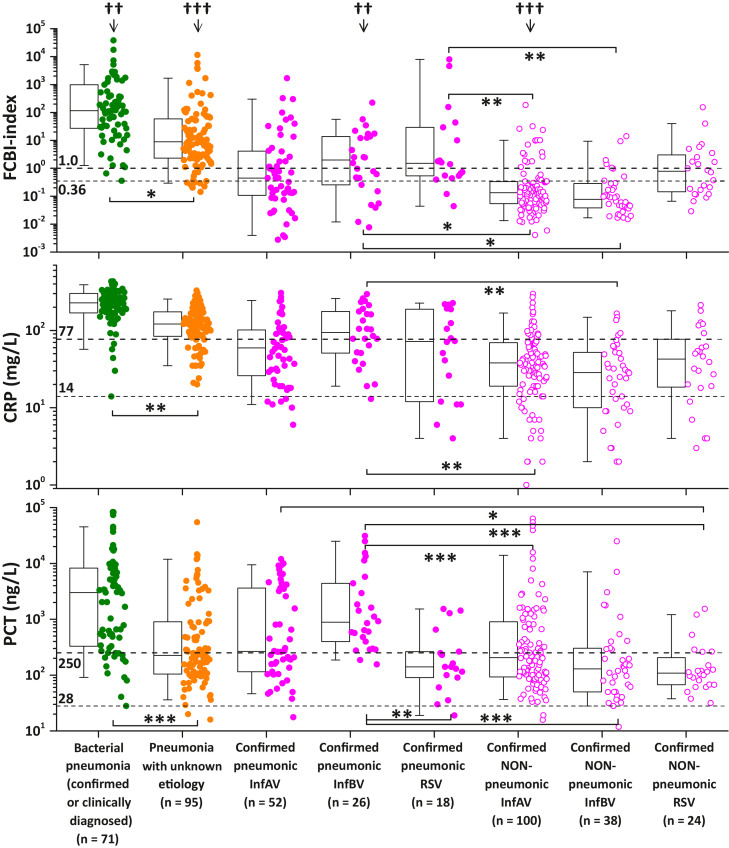
Distribution of the FCBI-index, CRP and PCT values in different types of respiratory tract infections (RTIs). The upper dotted horizontal line represents FCBI-index (1.0), CRP (77 mg/L), and PCT (250 ng/L) cutoff values for detecting bacterial infection. The Kolmogorov-Smirnov test showed that the FCBI-index data was not normally distributed. Bonferroni corrected p-values of post-hoc pairwise between group comparisons after Kruskal-Wallis (one-way ANOVA on ranks) test are completely presented within the virally infected subgroups only (for the complete matrix of significant p-values, see Supplementary Table 8), *p < 0.05, **p < 0.01, ***p < 0.001. ††p < 0.01 and †††p < 0.001 denote significantly different proportions of increased (greater than or equal to the above cutoff values) parameter values between tested bacterial infection markers by chi-square test. Box plot statistics (25th and 75th percentiles, and median value) with the 5th and 95th percentile (whiskers) values are shown. The lower dotted horizontal line represents the minimum FCBI-index (0.36), CRP (14 mg/L), and PCT (28 ng/L) values (additional cut-off values in the text) of patients with bacterial pneumonia. InfAV: Influenza A virus, InfBV: Influenza B virus, RSV: Respiratory syncytial virus. Only the data of the three biggest viral infection subgroups (infA, InfB and RSV; n = 258) are shown.

**Fig. 8:**
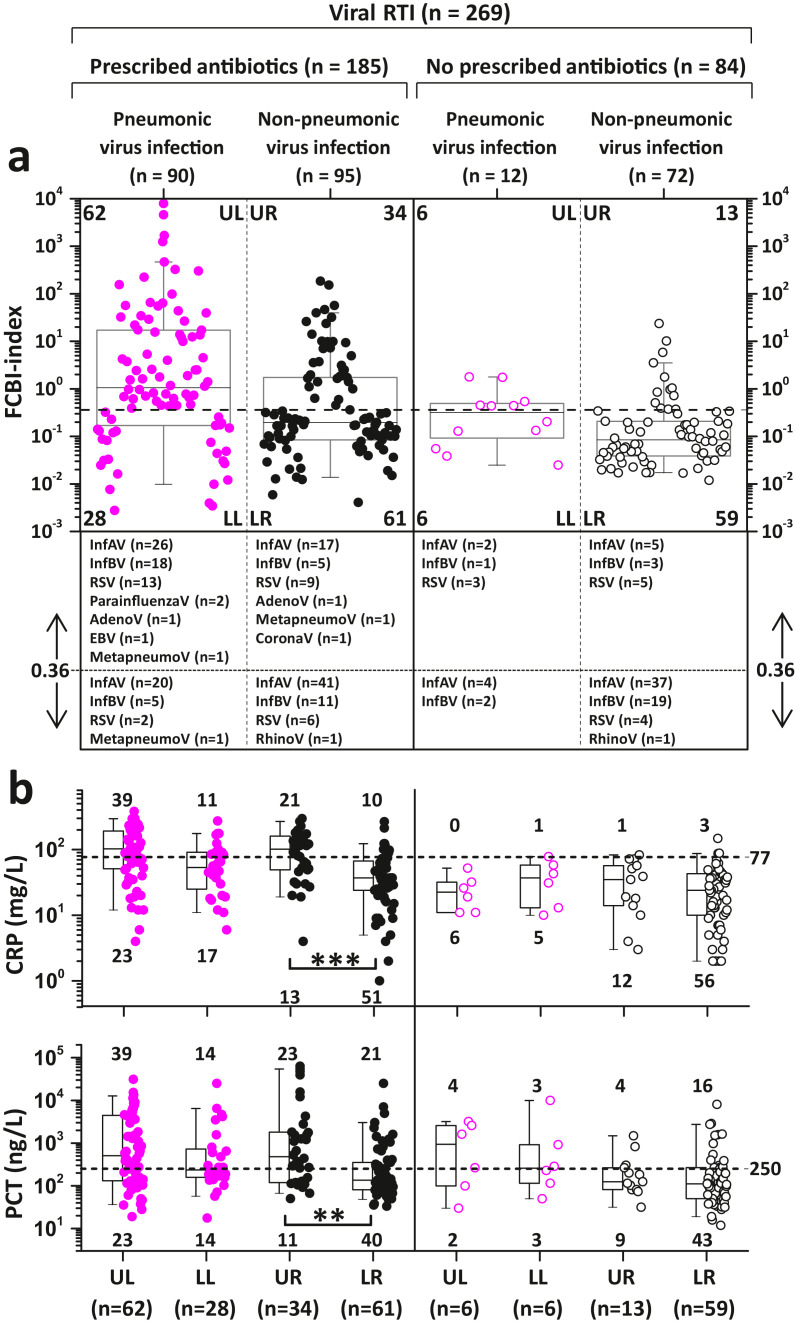
Antibiotic prescription and FCBI-index distribution among patients with viral respiratory tract infections (RTIs). (a) The dotted horizontal line represents the RTI-specific cutoff value of 0.36 for detecting possible bacterial coinfection (FCBI-index ≥ 0.36). UL, LL, UR, and LR stand for upper left, lower left, upper right, and lower right quarters, respectively. Comparative quarter-specific viral etiologies are presented at the bottom of the graphed data distribution. (b) Distribution of CRP and PCT values above and below the cutoff values of 77 mg/L and 250 ng/L, respectively, within the FCBI-index based UL, LL, UR, and LR quarters (presented in a). The Kolmogorov-Smirnov test showed that the CRP and PCT data was not normally distributed. Bonferroni corrected p-values of post-hoc pairwise between group comparisons of upper and lower quarters were determined after Kruskal-Wallis (one-way ANOVA on ranks) test, **p < 0.01, ***p < 0.001. (a and b) Box plot statistics (25th and 75th percentiles, and median value) with the 5th and 95th percentile (whiskers) values are shown. InfAV: Influenza A virus, InfBV: Influenza B virus, RSV: Respiratory syncytial virus, RhinoV: Rhinovirus, MetapneumoV: Metapneumovirus, EBV: Epstein-Barr virus, CoronaV: Coronavirus, AdenoV: Adenovirus

Sixty patient samples were excluded because of deficiencies in the quantitative receptor analysis (Fig. 1). We analyzed clinical data using the SPSS v25.0 (IBM, USA) software package. Data were presented as means ± SD (Supplementary Tables 4, 6 and 7) or plotted as individual data points with (Figs. 3, 4, 5, 6, 7 and 8, and Supplementary Figs. 3, 4, 5, 6 and 7) or without (Supplementary Figs. 8 and 9) box plot statistics, with 5th (whisker), 25th, 50th (median), 75th, and 95th (whisker) percentile values. Data were tested for normality using the Kolmogorov-Smirnov test. We compared continuous variables using the Kruskal-Wallis (one-way ANOVA on ranks) test with Bonferroni post hoc tests for multiple testing. Categorical variables were compared using a cross-tabulation test with chi-square (χ2) analysis. The efficiency of the FCBI-index, CRP and PCT in differentiating between confirmed bacterial and viral infections was evaluated using receiver operating characteristic (ROC) curve analysis. A 95% confidence level was used to calculate confidence intervals (95% CI). Two-sided probability (p)-values of 0.05 or less were considered to indicate statistical significance.

### 2.8 Sample-size estimation

The following formula was used to estimate necessary sample size for reliable differentiation between bacterial and viral infections:

Necessary sample size (n) = [(Z-score)^2^ × StdDev × (1-StdDev)]/(Margin of error)^2^

In calculations, we chose a 95% confidence level (Z-score = 1.96), 0.5 standard deviation and a margin of error (confidence interval) of +/- 5%: n = 1.96^2^ × 0.5 × 0.5/0.05^2^ = 384 samples

However, from our earlier similar cohort studies we have learned that the proportion of the patients with confirmed diagnosis of infection (bacterial or viral) from all febrile patients with suspected infection is about 40%[9,12]. Therefore, the final necessary sample size can be calculated as follows: n=384/0.4=960 samples. In the present study, totally 901 patient samples were collected, from which 484 (54%) got confirmed diagnosis (193 bacterial+291 viral).

### 2.9 Role of Funders

This study has been financially supported by Turku University Hospital (governmental EVO grant 13900) and The Finnish Medical Foundation (grant 4084/2014).

The funding sources played no role in the study design, data collection, data analysis, interpretation, writing of the report, and the decision of paper submission.

## 3 Results

In this comparative clinical study, we developed, evaluated and applied a novel flow cytometric method, a mathematical algorithm called the FCBI-index (Fig. 2; Supplementary Figs. 1-5), to predict bacterial infection in a cohort of 841 febrile patients suspected to have an infection (Fig. 1). Three main FCBI-index cutoff values were specified in order to ensure the accurate detection of febrile bacterial infection, and to decide if empiric antibiotic treatment is appropriate. All 193 patients with microbiologically confirmed bacterial infection had an FCBI-index ≥ 0.18, all 71 patients with bacterial lower respiratory tract infection (RTI, pneumonia) had an FCBI-index ≥ 0.36, and all 51 healthy controls had an FCBI-index < 1.0 (Fig. 3a). Among patients with confirmed bacterial infection, a majority (88%, 7/8) of the cases with an FCBI-index < 1.0 were bacteremic. Within the heterogeneous group of 254 patients with a fever of other or unknown origin, the proportion of patients having increased FCBI-index values varied from 31% in patients with rheumatic disorders to 85% in patients with pneumonic symptoms (Supplementary Fig. 6).

Used flow cytometry (FCM1 or FCM2) did not have statistically significant influence on the final FCBI-index distributions (Fig. 4).

Neither age nor gender have any influence on the detection of bacterial infection using the FCBI-index (Fig. 5).

### 3.1 The distribution of FCBI-index, CRP, and PCT values in different bacterial infections

There were two main observations supporting the applicability of the FCBI-index as a potent bacterial infection marker. Firstly, the area under the receiver operating characteristic (ROC) curve (AUC) statistic showed that the FCBI-index had an AUC of 0.901 (95% CI: 0.874–0.927), superior to that of CRP (AUC = 0.815, 95% CI: 0.777–0.853) and PCT (AUC = 0.715, 95% CI: 0.669–0.762) in distinguishing between microbiologically confirmed bacterial (n = 193) and viral (n = 291) infections (see the table associated with Fig. 3b). Secondly, the distribution of FCBI-index values was the same across the studied bacterial infection subgroups, whilst 96% (264/275) (95% CI: 94–98%) of all FCBI-index values within these subgroups were above the cutoff value of 1.0 (Fig. 3c). The corresponding numbers for CRP and PCT were 82% (225/275) (95% CI: 77–87%) and 75% (205/275) (95% CI: 69–79%), respectively. According to the Pearson chi-square analysis, the three compared methods had significantly different percentages of patients with increased parameter values in all three bacterial infection subgroups (Fig. 3c).

Procalcitonin, a generally accepted marker of sepsis [26], was increased in 89% (95% CI: 83–95%) of the bacteremic patients. Corresponding numbers for CRP and the FCBI-index were 84% (95% CI: 77–91%) and 94% (95% CI: 90–98%), respectively. However, in some local microbiologically confirmed (pyelonephritis and gastroenteritis) and clinically diagnosed probable (erysipelas) bacterial infections (Fig. 6), a significantly high proportion (40–60%) of patients had low PCT values (< 250 ng/L), while a significantly high proportion (95%) of the same patients had increased FCBI-index values. The observed low PCT level in local bacterial infections is in line with the meta-analysis of 12 studies in 2408 patients by Kamat et al., concluding that PCT level has an overall sensitivity of 55% for detecting bacterial infection and is unlikely to provide reliable evidence either to mandate or withhold administration of antibiotics in patients with pneumonia [27]. Based on presented evidences, it can be concluded that FCBI-index and CRP are general markers of bacterial infection whereas serum PCT is useful only in predicting bacteremia in critically ill febrile patients.

The value distributions of any of the three bacterial infection markers did not differ significantly between Gram-negative and Gram-positive bacterial infections (Supplementary Fig. 7).

Of 193 patients with microbiologically confirmed bacterial infections, 11 also had concomitant confirmed viral infection, of which 10 (91%) had an FCBI-index ≥ 1.0 (Supplementary Fig. 8), indicating that simultaneous viral infection does not have a significant influence on the detection of bacterial infection using the FCBI-index.

### The distribution of FCBI-index, CRP, and PCT values in different RTIs

Since there were not statistically significant differences in the pairwise comparisons of FCBI-index, CRP and PCT values between bacteremic (n = 26), microbiologically confirmed local (n = 13), and clinically diagnosed probable (n = 32) bacterial pneumonia subgroups (Fig. 6)[p-values for bacteremic vs local pneumonia, bacteremic vs clinical pneumonia, and local vs clinical pneumonia pairwise comparisons were 1.0, 1.0, and 0.165 for CRP, 1.0, 1.0, and 1.0 for PCT, and 1.0, 1.0, and 1.0 for FCBI-index, respectively], it was justified to combine the patients from these subgroups to form a new bacterial pneumonia subgroup (confirmed or clinically diagnosed, n = 71) presented in Fig. 3a and 7.

Pairwise between group comparisons showed that the FCBI-index and CRP values of the bacterial pneumonia subgroup were statistically significantly increased when compared with the six virally infected RTI subgroups and patients having pneumonia with unknown etiology (Fig. 7 and Supplementary Table 8). The same holds true for the PCT data, except that bacterial pneumonia subgroup and patients with pneumonic influenza B virus infection did not differ significantly from each other. In patients having pneumonia with unknown etiology, the FCBI-index and CRP values (but nor PCT values) were statistically significantly increased when compared to patients with non-pneumonic viral RTIs and pneumonic influenza A virus infection.

The FCBI-index value was increased (≥ 1.0) in 97% (95% CI: 93-100%) of the patients with bacterial pneumonia and in 87% (95% CI: 79-100%) of the patients with pneumonia with unknown etiology, while the corresponding percentages were 93% (95% CI: 87-99%) and 79% (95% CI: 70-100%) for CRP, and 79% (95% CI: 70-100%) and 44% (95% CI: 32-56%) for PCT, respectively. On the other hand, an increased PCT value was observed in 93% (95% CI: 83-100%) of the patients with pneumonic influenza B infection and in 46% (95% CI: 36-56%) of the patients with non-pneumonic influenza A infection, while the corresponding percentages were 65% (95% CI: 47-83%) and 21% (95% CI: 13-29%) for CRP, and 54% (95% CI: 35-73%) and 17% (95% CI: 10-24%) for the FCBI-index, respectively. According to the Pearson chi-square analysis, the above-mentioned differences in the percentages of patients with increased parameter values between the three compared methods were significantly different (Fig. 7).

### 3.2 Use of the minimum value of the bacterial pneumonia group as an alternative cutoff value in RTIs

The positive effect of the wide dynamic range of the method on bacterial-viral differential diagnostics could be seen when the minimum value of the bacterial pneumonia group was used as an alternative cutoff value (0.36 for FCBI-index, 14 mg/L for CRP, and 28 ng/L for PCT) in patients with RTI (Fig. 7).

With the FCBI-index method, possessing the widest dynamic range of eight decades, 43% (115/269, 95% CI: 37–49%) of the patients with viral RTI had an FCBI-index ≥ 0.36, indicating the possibility of bacterial coinfection. This percentage is in line with the meta-analysis of 27 prospective studies by Klein et al., showing that between 2% and 65% of the patients with laboratory-confirmed influenza had a bacterial coinfection [28]. In addition, Falsey et al. reported in their study of 771 patients that bacterial coinfection (diagnosed by a positive bacterial assay result [n = 64] or a serum PCT level of ≥ 250 ng/L [n = 72]) was found in 39% of 348 viral respiratory tract infections requiring hospitalization [24]. In the presented study, the proportion of potential bacterial coinfections in pneumonic and non-pneumonic viral RTI was 66% and 27%, respectively. It was also noticed that the proportion of potential bacterial coinfections differed significantly depending on viral etiology: Influenza A virus: 33% (50/152, 95% CI: 26–40%), Influenza B virus: 42% (27/64, 95% CI: 30–54%), and Respiratory syncytial virus: 71% (30/42, 95% CI: 57–85%), χ2 (df2) = 20.148, p < 0.001.

In the case of PCT and CRP, which have a dynamic range of four and three decades, the use of the alternative cutoff values of 28 ng/L and 14 mg/L led to the suspiciously high proportion of probable bacterial coinfection: 98% and 83%, respectively.

Of those 16 virally infected patients with clinical signs of bacterial pneumonia (lobar pneumonia detected by chest radiograph or pneumonia with CRP higher than 200 mg/L), 94% (15/16) had an increased (≥ 0.36) FCBI-index value (range: 1.40–7951, median: 22).

### 3.3 Antibiotic prescription and FCBI-index distribution among patients with viral RTI

In retrospect, the prevalence of symptom-based empiric antibiotic prescription for the patients with febrile viral RTI was 69% (185/269, 95% CI: 63–75%) in this study (Fig. 8a).

Of the 185 antibiotic-treated patients, only 52% (62 pneumonic in the upper left quarter [UL] and 34 non-pneumonic in the upper right quarter [UR] in Fig. 8a) had increased FCBI-index values (≥ 0.36), suggesting bacterial coinfection. Among the 86 patients not treated with antibiotics, an increased FCBI-index value was found in 23% (6 pneumonic [UL] and 13 non-pneumonic [UR] in Fig. 8a) of the cases, raising the total number of patients with probable bacterial coinfection (according to the FCBI-index method) to 115. Serum CRP and PCT followed only partly the FCBI-index-based distribution of patients into UL, LL, UR and LR quarters, respectively (Fig. 8B).

In the 22 patients with viral non-RTI, both the prevalence of the symptom-based empiric antibiotic prescription and the proportion of patients with an increased FCBI-index value (≥ 0.18) was 77% (Supplementary Fig. 9). Puumala virus infection was a special case among the other viral infections, in that all 10 patients suffering from it had an increased PCT value (range: 689–9630 ng/L, median: 1250 ng/L) and seven also had an increased FCBI-index value (range: 3.57–1744, median: 57), both reflecting the severe clinical symptoms of this viral infection. Ninety percent of the Puumala virus-infected patients were treated with empiric antibiotics before the diagnosis was confirmed.

## 4 Discussion

Clinicians are often tempted to prescribe symptom-based antibiotics “just to be on the safe side” to avoid the onset of a severe and possibly life-threatening bacterial infection [29]. However, besides being ineffective, treating viral illnesses or non-infective causes of inflammation with inappropriate antibiotics contributes to the development of resistance, increasing medical costs. Therefore, the development of rapid and accurate bacterial infection markers is a necessity to avoid inappropriate antibiotic prescriptions.

We have developed a novel and simple flow cytometric-based bacterial infection marker, the FCBI-index, to detect febrile bacterial infections reliably within 30 minutes. The method is based on the quantitative analysis of host markers CD35, CD64, and CD329 receptors on neutrophils and lymphocytes. The fourth member of the antibody kit, an anti-human MHCI antibody, was exploited as a pan-leukocyte marker in order to sort fluorescent leukocytes from non-fluorescent debris. The reagent cost per test, consisting mainly of presented antibody kit, is only about 5.5 € (6.6 $).

The incubation of the whole blood sample with the receptor-specific antibodies, red blood cell lysis, and flow cytometric run were carried out one after another in the same test tube without any time-consuming washing steps. This differs from our previous studies, where antibody incubation was performed with isolated leukocytes. The FCBI-index test requires only a minimal amount of the whole blood sample (22 μL), making it possible to utilize a fingertip blood sample as the starting material [30]. A novel clinical study is in development to test this hypothesis.

In the data handling process, the incorporation and conversion of the raw receptor expression data into a single FCBI-index value was performed using the mathematical bacterial infection algorithm. In this study, the FCBI-index value was calculated by substituting the obtained receptor-specific MFI data into the bacterial infection algorithm manually, but it should be relatively easy to create flow cytometry data analysis software for automated FCBI-index calculation.

An incomplete screening of the bacterial pathogens among virally infected febrile patients, as well as the lack of day-to-day follow-up of how the FCBI-index values vary during the course of the infection, were the most noteworthy study design-related limitations of the presented research. We plan to conduct a future clinical follow-up study addressing the above issues with a limited patient cohort. In particular, patients diagnosed with inflammatory conditions (such as autoimmune diseases, asthma and gout), whose clinical presentation and FCBI-index distribution seemed to be quite similar to that of bacterial infection (Supplementary Fig. 6), as well as immunocompromised patients will be a subject of special attention when selecting control groups for these future studies. Another limitation of the method itself is that FCBI-index-based determination of the exact type of bacterium (or virus) causing the infection is impossible. Therefore, the use of the FCBI-index method as a preliminary test preceding and directing: a) confirmatory identification of pathogens via PCR, antigen detection or enzyme-linked immunosorbent assay (ELISA) tests, and b) empiric antibiotic treatment, for example, would be the best strategy to achieve optimal economic and clinical benefits.

Of course, several studies of independent research groups will be needed in the future to confirm our observation that the used flow cytometry does not have significant influence on FCBI-index based distinguishing between bacterial and viral infections. When calculating FCBI-index values in future clinical studies, we recommend primarily using predetermined constant denominator (3.6, 3 and 7) and power (3, 1.6 and 1.4) values in the algorithm. However, if one wants to make some changes to these constant values, they must be minor in scale and should be performed gradually. For example, if the power value of 3.1 is used instead of 3, then the final FCBI-index value will be about 0.9-1.3-fold compared with an original one, depending on CD35 RATIO value of course. On the other hand, if the denominator of 3.5 is used instead of 3.6 when calculating CD35 RATIO, then the final FCBI-index will be 1.088-fold compared with an original one.

Based on the presented evidence, we can speculate that the FCBI-index value in combination with the clinical course of the febrile illness can be utilized to give reliable recommendations for or against the initiation of empiric antibiotic therapy. From an ethical standpoint, the risk of a life-threatening bacterial infection should always be eliminated. Therefore, the routine use of a single FCBI cutoff value does not necessarily lead to the best balance between cost-effectiveness and treatment outcome, the latter being the first priority. Instead, it may be more beneficial to employ three FCBI cutoffs to ascertain the appropriate antibiotic prescription using the FCBI-index (Fig. 3a). Two cutoff values, FCBI-index ≥ 0.36 and ≥ 0.18, are workable as a threshold for starting empiric antibiotic treatment in febrile patients with or without RTI symptoms, respectively (Supplementary Fig. 10). In the case that the FCBI-index value stays below 0.18 (patients with non-RTI symptoms) or 0.36 (patients with RTI symptoms), the empiric antibiotic should be prescribed only if the bacterial etiology has been confirmed. Once antibiotic treatment has been initiated, it should not be routinely stopped until fever and other specific clinical symptoms of infection have decreased, and the elevated FCBI-index value has recovered below the third cutoff value of 1.0, indicating resolution of bacterial infection. To confirm resolution of bacterial infection, we suggest that FCBI-index determination should be repeated at least every 24 hours. In the case that the FCBI-index remains high in spite of the drug therapy, then switching to another antibiotic might be considered. The evaluation of the presented recommendations will be the subject of the future studies.

Retrospectively, we can speculate that, if the antibiotic treatment-related course of actions described above had been performed in the patients with RTI symptoms in this study, then an immediate empiric antibiotic would have been prescribed for 115 (43%) of the 269 patients with a viral RTI, 38% less than the actual 185 prescriptions given.

We conclude that the proposed FCBI-index method, particularly when associated with inexpensive, portable and easy-to-use flow cytometer, might be a potent part of antimicrobial stewardship measures in hospitals and health centers, helping physicians to decide whether empiric antibiotic treatment is necessary or not.

## Contributors

J.N. and P.J-K. contributed to project conceptualisation and study design. J.N. contributed to flow cytometric methodology and literature search, J.N., U.H. and P.J-K. contributed to sample collection and investigation of samples, P.J-K. and U.H. contributed to administration of patient records, J.N. and P.J-K. contributed to data analysis and data visualisation. J.N. J.O. and P.J-K. provided project supervision. All authors contributed to writing and editing. All authors read and approved the final version of the manuscript. All authors have verified the underlying data.

## Data sharing statement

The authors declare that the main data supporting the findings of this study are available within the article and its Supplementary Information file. Extra data are available from the corresponding author upon request (jarnuu@utu.fi).

## Declaration of Competing Interest

P.J-K declares consulting fees from MSD and GSK, participation on an advisory board for MSD and GSK, and stock in Orion, all outside of the submitted work. All other authors declare that they have no competing interests.

## Appendix Supplementary materials

Image, application 1
