# Supplementary material for: Rapid detection of bacterial infection using a novel single-tube, four-colour flow cytometric method: Comparison with PCT and CRP
Source: eBioMedicine. 2021 Nov 26;74:103724. doi: 10.1016/j.ebiom.2021.103724 (PMC8633870; doi:10.1016/j.ebiom.2021.103724)
Supplement: Supplementary file 1 [file mmc1.pdf]

Supplementary Materials for

Rapid detection of bacterial infection using a novel single-tube, four-color flow cytometric method: Comparison with PCT and CRP

Supplementary:

- ***Fig. 1:*** The four stages of the quantitative flow cytometry-based receptor assay.
- ***Fig. 2:*** An illustration of how to set subpopulation-specific gates around leukocytes, neutrophils and lymphocytes during a flow cytometric run.
- ***Fig. 3:*** Flow cytometric data analysis and calculation of CD35, CD64 and CD329 ratios.
- ***Fig. 4:*** Receptor-specific RATIO to INDEX conversions and calculation of the FCBI-index value.
- ***Fig. 5:*** Constant denominator (3.6, 3 and 7; receptor specific cutoff values for RATIOs) and power (3, 1.6 and 1.4) values in the FCBI-index algorithm were obtained by iteration (trial and error method).
- ***Fig. 6:*** Distribution of the FCBI-index value in 254 febrile patients with the fever of unclear etiology.
- ***Fig. 7:*** Distribution of FCBI-index, CRP and PCT values in patients with bacteremia or confirmed local bacterial infection caused by Gram-positive or Gram-negative bacteria.
- ***Fig. 8:*** Distribution of FCBI-index values in patients with simultaneously confirmed bacterial and viral infections, and confirmed viral infection with asymptomatic bacteriuria.
- ***Fig. 9:*** Antibiotic prescription and FCBI-index distribution among patients with viral non-RTI infections.
- ***Fig. 10:*** Possible exploitation of the three major FCBI-index cutoff values of 0.18, 0.36 and 1.0 in antibiotic prescription.

- ***Table 1:*** Detected bacterial pathogens causing the confirmed infectious diseases.
- ***Table 2:*** Detected viral pathogens causing the confirmed infectious diseases.
- ***Table 3:*** Group-specific antimicrobial prescriptions.
- ***Table 4:*** Demographic and clinical characteristics of the patients at baseline.
- ***Table 5:*** Characteristics of the monoclonal fluorescent-labelled antibodies used in the study.
- ***Table 6:*** Reproducibility test number 1.
- ***Table 7:*** Reproducibility test number 2.
- ***Table 8:*** Complete matrix presenting significant p-values of pairwise comparisons between different respiratory tract infection subgroups for Fig. 7 data.

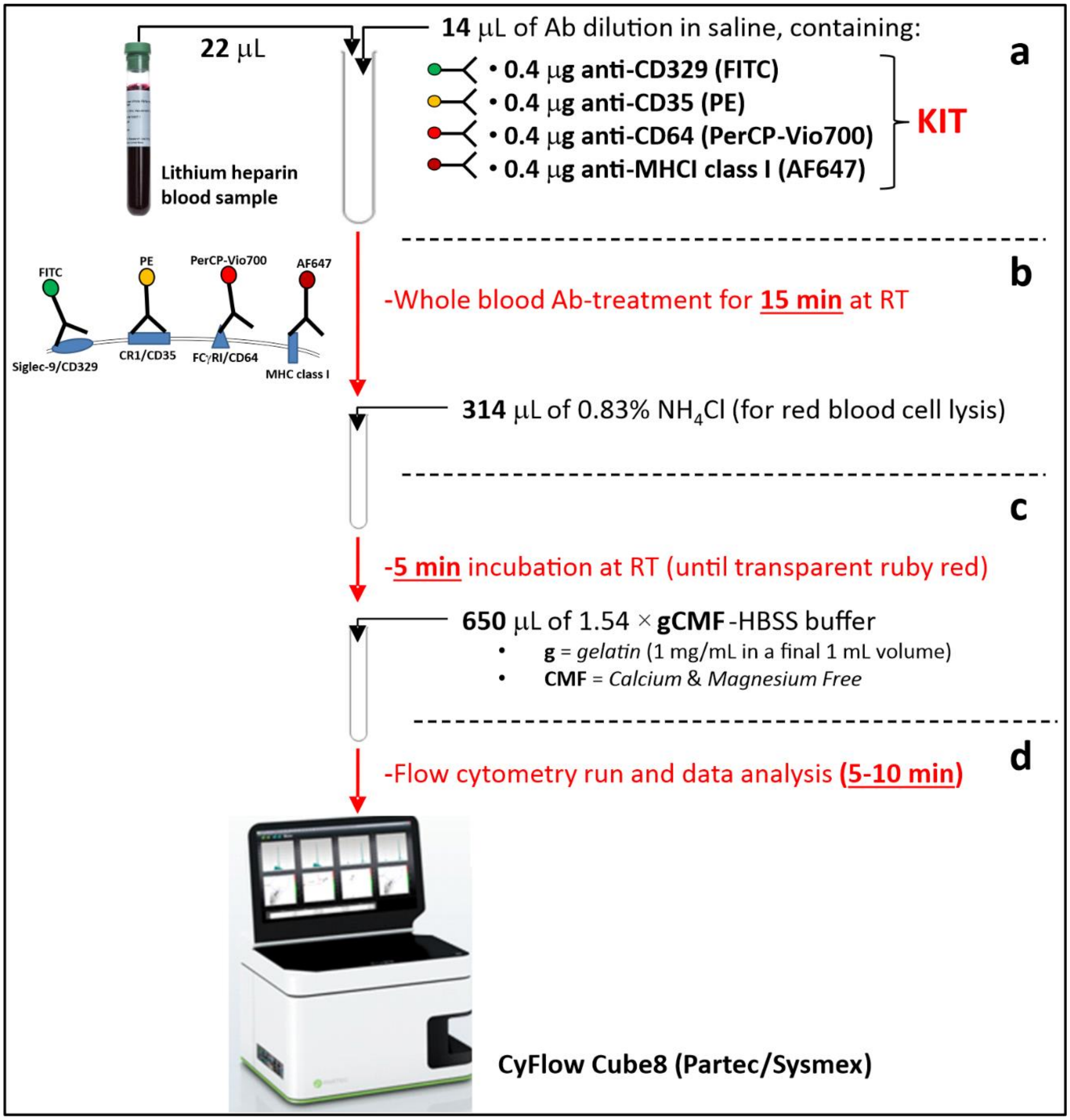

Supplementary Fig. 1. The four stages of the quantitative flow cytometry-based receptor assay. (a) Mixing of whole blood sample and antibody dilution (kit) containing 0.4 μg of each monoclonal antibody (anti-human CD35, CD64, CD329, and MHC class I) in one sample tube. **(b)** Whole blood antibody treatment, during which the monoclonal antibodies bind to their receptors. **(c)** Red blood cell lysis. **(d)** Flow cytometric run. Note that all stages are performed consecutively in one test tube without any time-consuming washing and centrifugation steps.

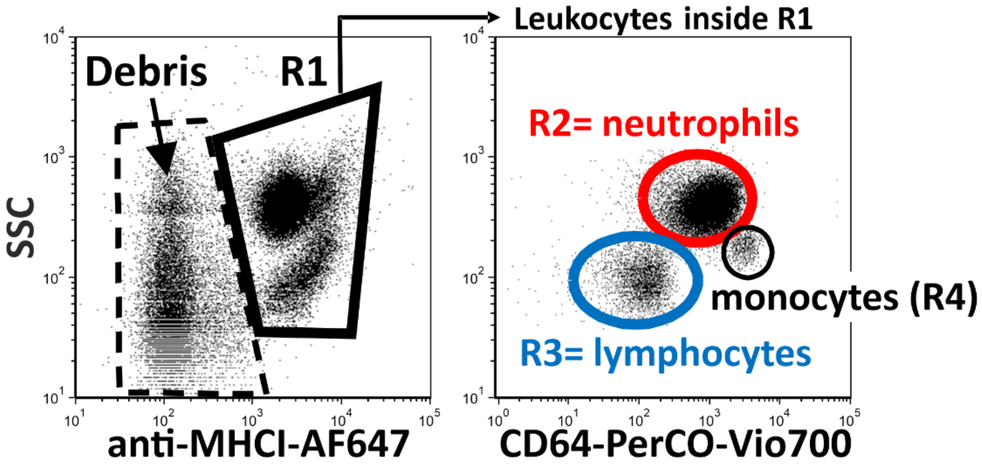

Supplementary Fig. 2. An illustration of how to set subpopulation-specific gates around leukocytes (R1), neutrophils (R2), and lymphocytes (R3) during a flow cytometric run. In the first phase of the run, the leukocyte population (granulocytes and agranulocytes) within gate R1 was gated out from the SSC log/MHCI log bivariate histogram (left). As MHCI is expressed on all leukocytes, its function in the kit is to separate debris from the intact leukocytes. In the second phase of the run, the gates R2 and R3 were placed around neutrophil and lymphocyte populations, respectively, in the SSC log/CD64 log bivariate histogram containing leukocytes inside gate R1 (right). The data was collected until the stop count of 1000 monocytes inside the monocyte gate R4 was reached (about 5000–20000 leukocytes/run in total).

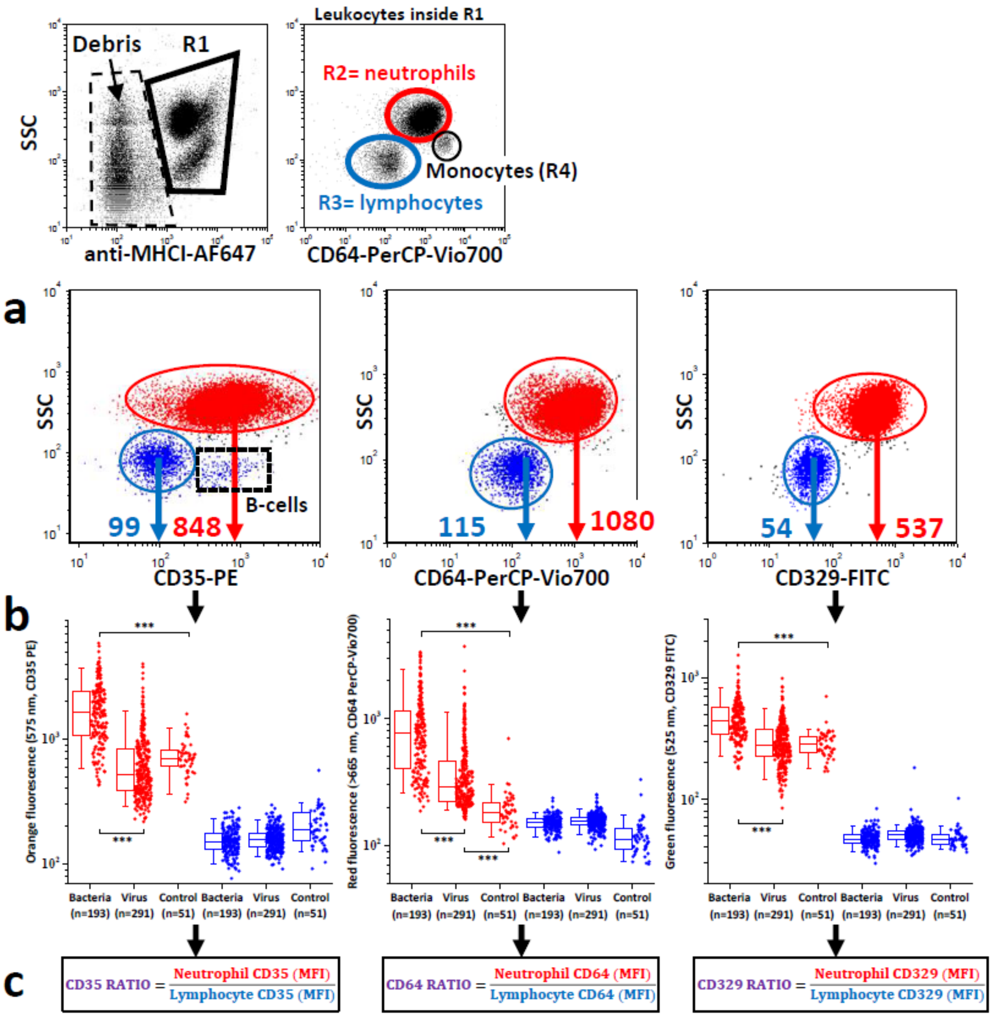

Supplementary Fig. 3. Flow cytometric data analysis and calculation of CD35, CD64 and CD329 ratios. (a) For the purpose of data analysis, three additional bivariate histograms, namely SSC log/CD35 log, SSC log/CD64 log, and SSC log/CD329 log were built, showing mean fluorescence intensities (MFI, correlating with the number of receptors on the cell surface) of gated neutrophil (red) and lymphocyte (blue) populations. In the SSC log/CD35 log histogram, B lymphocytes (B cells) expressing CD35 were left outside the lymphocyte gate. **(b)** Receptor-specific MFI values of neutrophil (red) and lymphocyte (blue) populations in confirmed bacterial (n = 193) and viral infections (n = 291) and in healthy controls (n = 51). The Kolmogorov-Smirnov test showed that the receptor expression data was not normally distributed. The Bonferroni corrected p-values of post-hoc pairwise between group comparisons were determined after the Kruskal-Wallis (one-way ANOVA on ranks) test, ***$p < 0.001$. Box plot statistics [25th and 75th percentiles (the bottom and top of the box), and median value (horizontal line in the middle of the box)] with the 5th and 95th percentile (whiskers) values are shown. **(c)** In order to calculate so-called RATIO-values for CD35, CD64 and CD329, the receptor-specific MFI value of the neutrophil population was divided by that of lymphocyte population. As cell type-specific MFI values act as each other’s internal controls on an individual flow cytometric run, the calibration bead-based day-to-day normalization of MFI raw data is unnecessary when calculating CD35, CD64 and CD329 RATIOs.

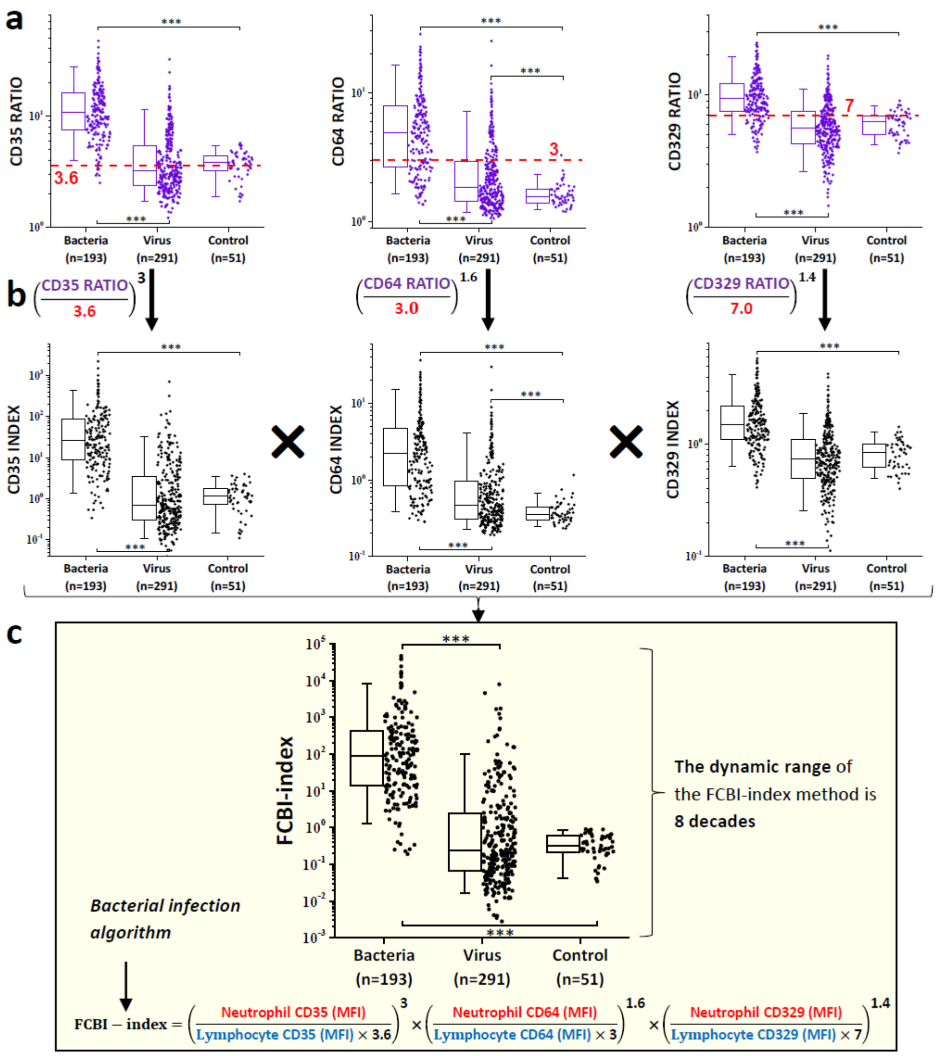

Supplementary Fig. 4. Receptor-specific RATIO to INDEX conversions and calculation of the FCBI-index value. (a) Distribution of CD35, CD64 and CD329 RATIO values in confirmed bacterial (n = 193) and viral (n = 291) infections and in healthy controls (n = 51). **(b)** Receptor-specific RATIO to INDEX conversions were performed using conversion formulas. The receptor-specific constant cutoff values (3.6 for CD35 RATIO, 3 for CD64 RATIO, and 7 for CD329 RATIO) and powers (3 for CD35 RATIO, 1.6 for CD64 RATIO, and 1.4 for CD329 RATIO) used in the conversion formulas were obtained by iteration (trial and error method) in order to get the best differentiation between bacterial and viral infections using the final FCBI-index. **(c)** The FCBI-index was obtained by multiplying the CD35 INDEX, CD64 INDEX and CD329 INDEX values. ***The FCBI-index can also be obtained without intermediate calculation stages by using the bacterial infection algorithm, which incorporates receptor-specific RATIO and INDEX calculations into one mathematical formula*. (a, b and c)** The Kolmogorov-Smirnov test showed that the CRP and PCT data was not normally distributed. The Bonferroni corrected p-values of post-hoc pairwise between group comparisons were determined after the Kruskal-Wallis (one-way ANOVA on ranks) test, $^{**}p < 0.01$, $^{***}p < 0.001$. Box plot statistics (25th and 75th percentiles, and median value) with the 5th and 95th percentile (whiskers) values are shown.

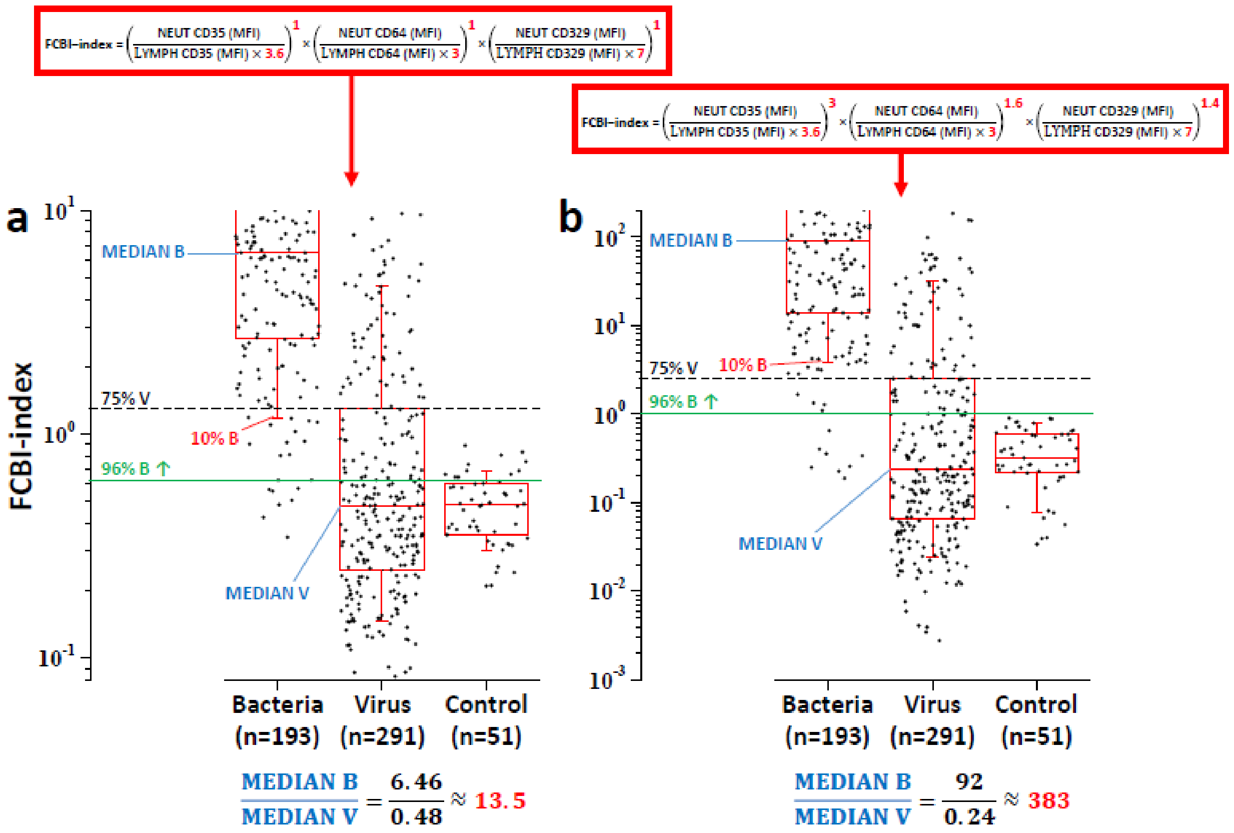

Supplementary Fig. 5. Constant denominator (3.6, 3 and 7; receptor specific cutoff values for RATIOs) and power (3, 1.6 and 1.4) values in the FCBI-index algorithm were obtained by iteration (trial and error method). (a) In the first phase of the iteration, constant denominators were set so that MEDIAN B/MEDIAN V-ratio was as high as possible (13.5). Optimal receptor-specific denominators were obtained in a following order: first CD35-dominator (3.6), then CD64-denominator (3), and finally CD329-denominator (7). While obtaining optimal denominators, all three receptor specific power values were set to one in the algoritm. **(b)** In the second phase of the iteration, after constant denominators were obtained and set into the algoritm, constant power values were set so that MEDIAN B/MEDIAN V-ratio was as high as possible (383). Optimal receptor-specific power values were obtained in a following order: first CD35 specific power (3), then CD64 specific power (1.6), and finally CD329-specific power (1.4). **(a and b)** 96% of the bacterial infection cases (96% B) are located above the green line (in b, the green line represents the final FCBI-index cutoff value of 1) and 75% of the viral infection cases (75% V) are located below the dotted black line. As a result of optimal iteration (in b), 100% of the control values are below the green line and 6% of the bacterial infection cases are below the 75% V-value (in a, however, only 78% of the control values are below the green line and 10% of the bacterial infection cases are below the 75% V-value). MEDIAN B; the median value of the bacterial infection group, MEDIAN V; the median value of the viral infection group, 10% B; a lower whisker, below which 10% of the bacterial infection cases are located.

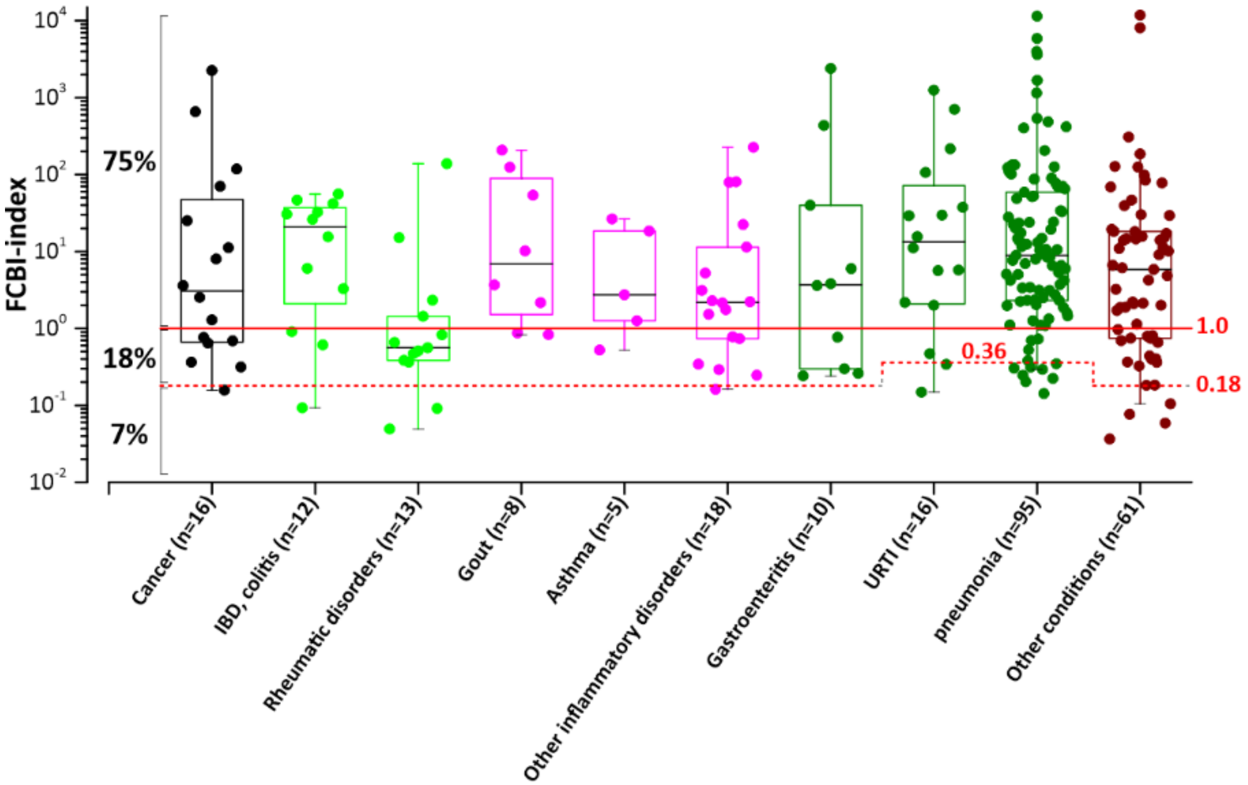

Supplementary Fig. 6. Distribution of the FCBI-index value in 254 febrile patients with fever of other or unknown origin. Subgroups of patients diagnosed with cancer (black), autoimmune (light green) and inflammatory (magenta) disorders, suspected infectious disease (olive green), and a miscellaneous collection of other conditions (wine red) are shown. The three major FCBI-index cutoff values (0.18, 0.36 for URTI and pneumonia, and 1.0) cited in this manuscript are presented as red horizontal (dotted and solid) lines. Percentages on the left indicate how FCBI-index values are distributed in relation to the used cutoff values. Box plot statistics (25th and 75th percentiles, and median value) with the 5th and 95th percentile (whiskers) values are shown. IBD: Inflammatory bowel disease, URTI: Upper respiratory tract infection.

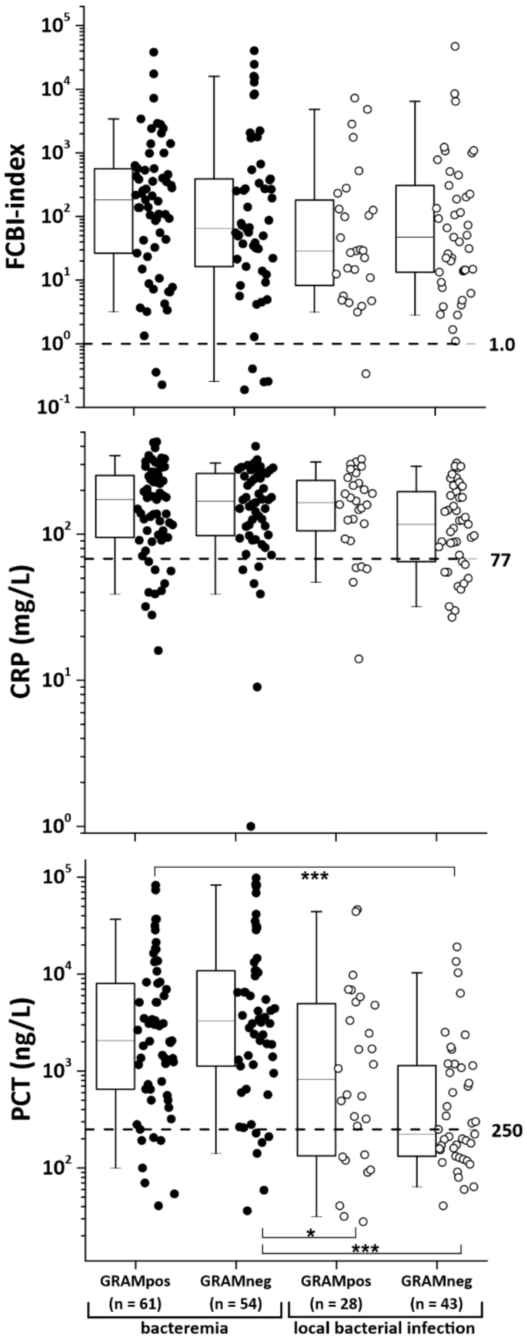

Supplementary Fig. 7. Distribution of FCBI-index, CRP and PCT values in patients with bacteremia (solid symbol) or confirmed local bacterial infection (open symbol) caused by Gram-positive or Gram-negative bacteria. Only the data of those 186 patients diagnosed with a single bacterial species is presented. The dotted horizontal lines represent the used cutoff value of FCBI-index (1.0), CRP (77 mg/L), and PCT (250 ng/L) when detecting bacterial infection. The Kolmogorov-Smirnov test showed that the presented data was not normally distributed. The Bonferroni corrected p-values of post-hoc pairwise between group comparisons were determined after the Kruskal-Wallis (one-way ANOVA on ranks) test, $^*p < 0.05$, $^{***}p < 0.001$. Box plot statistics (25th and 75th percentiles, and median value) with the 5th and 95th percentile (whiskers) values are shown.

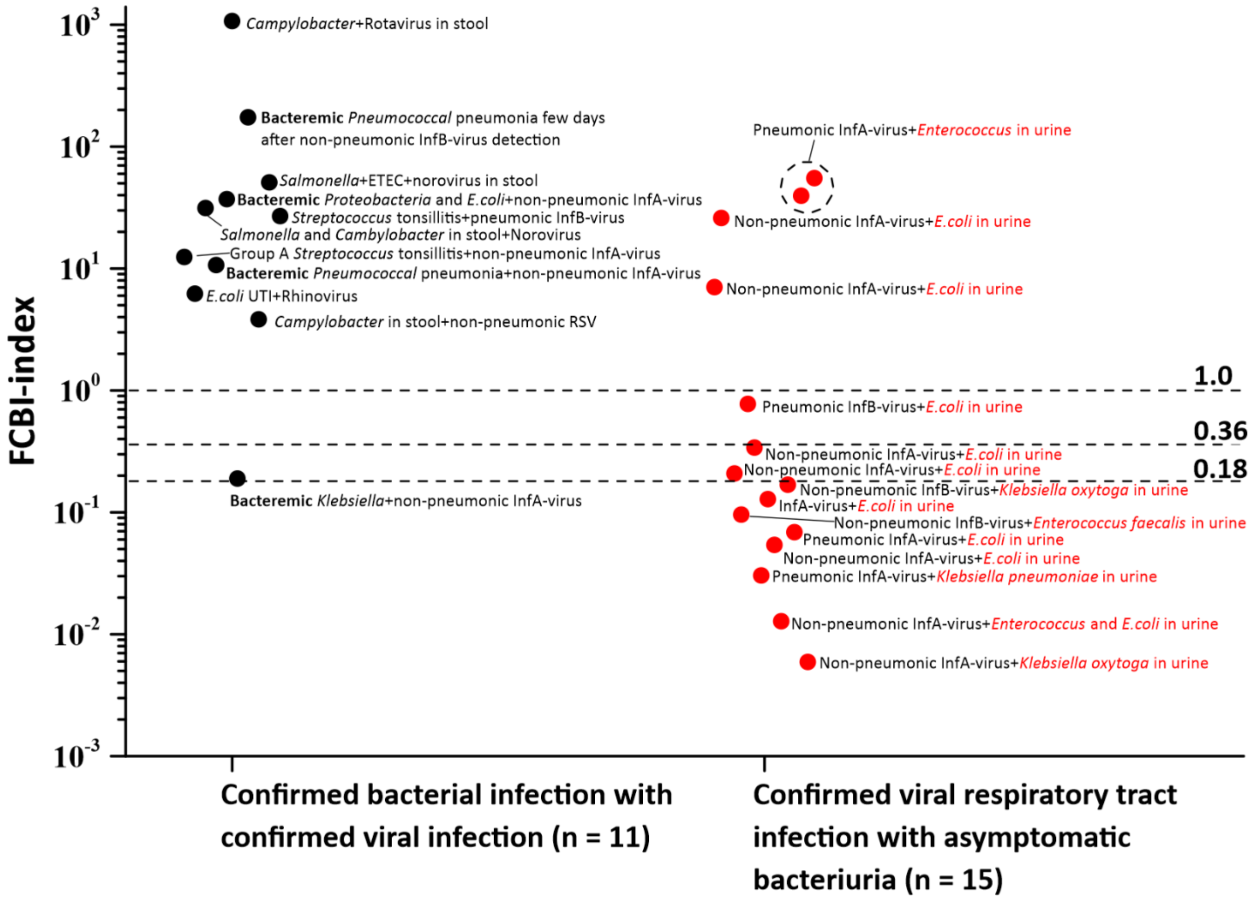

Supplementary Fig. 8. Distribution of FCBI-index values in patients with simultaneously confirmed bacterial and viral infections (black symbols), and confirmed viral infection with asymptomatic bacteriuria (red symbols). The dotted horizontal line represents the three FCBI-index cutoff values of 0.18, 0.36 and 1.0. UTI: urinary tract infection.

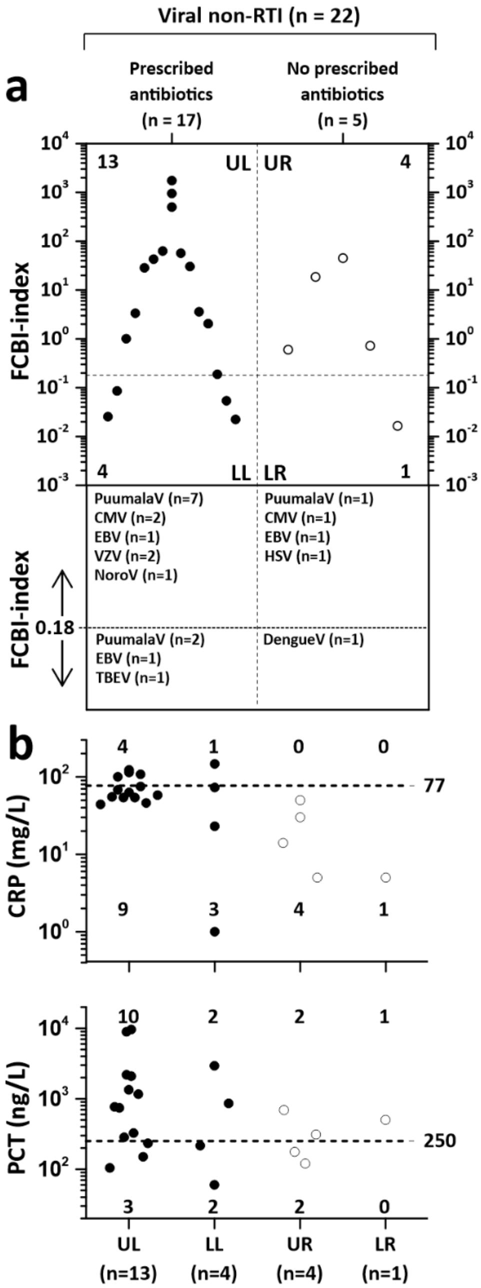

Supplementary Fig. 9. Antibiotic prescription and FCBI-index distribution among patients with viral non-RTI infections. (a) The dotted horizontal line represents the non-RTI-specific cutoff value of 0.18 for detecting possible bacterial coinfection (FCBI-index ≥ 0.18). Comparative quarter-specific viral etiologies are presented at the bottom of the graphed data distribution. UL, LL, UR and LR stand for upper left, lower left, upper right, and lower right quarters, respectively. **(b)** The distribution of quarter-specific CRP and PCT values above and below the cutoff values 77 mg/L and 250 ng/L, respectively, is indicated as absolute numbers. CMV: Cytomegalovirus, EBV: Epstein-Barr virus, VZV: Varicella zoster virus, TBEV: Tick-borne encephalitis virus, HSV: Herpes simplex virus.

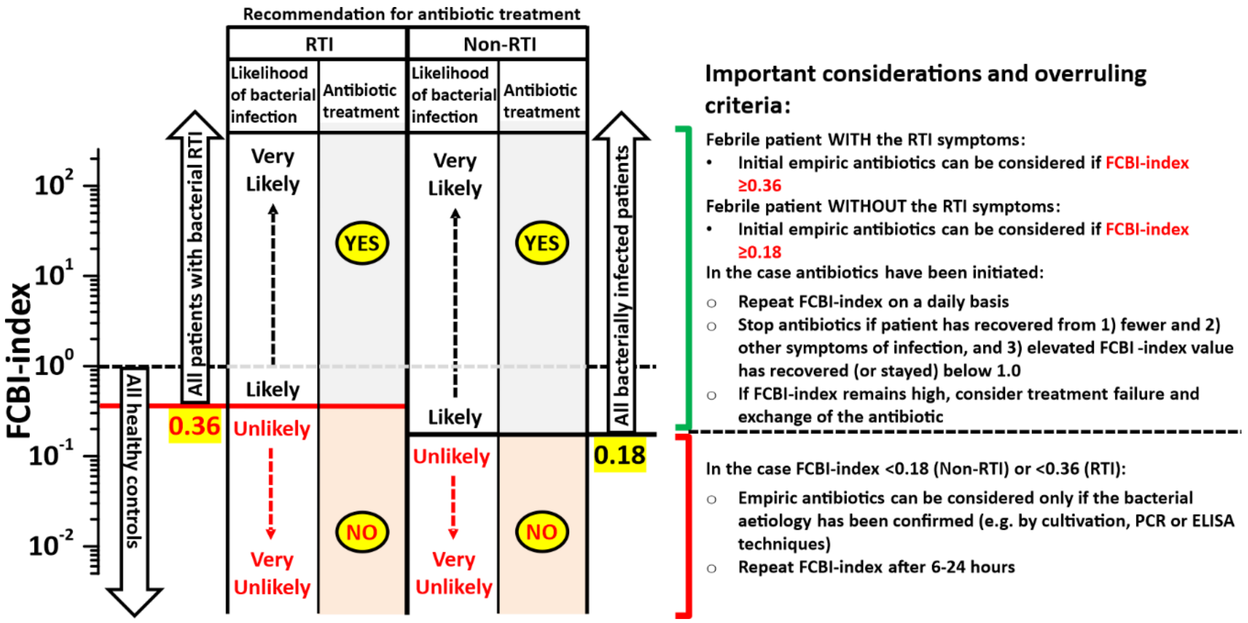

Supplementary Fig. 10. Possible exploitation of the three major FCBI-index cutoff values of 0.18, 0.36 and 1.0 in antibiotic prescription. RTI: Respiratory tract infection.

Supplementary Table 1. Detected bacterial pathogens causing the confirmed infectious diseases.

Diagnosis—(no.)	Pathogen(s)
**Bacteremia (49)**	*Staphylococcus* species (23, (*S.aureus 20*)), *Streptococcus* species (13), *Escherichia coli* (3), *Escherichia coli+Staphylococcus aureus* (1), *Escherichia coli+Proteus* (1), *Pseudomonas* species (2), *Enterococcus faecalis* (1), *Klebsiella* (1), *Klebsiella + Bacteroides* (1), *Pneumococcus* (1), *Salmonella Paratyphi* (1), *Yersinia enterocolitica* (1)
**Bacteremic UTI (43)**	*Escherichia coli* (38), *Escherichia coli + Enterococcus faecalis* (1), *Klebsiella pneumonia* (2), *Pneumococcus* (1), *Pseudomonas* (1)
**Bacteremic pneumonia (26)**	*Pneumococcus* (15), *Streptococcus pneumonia* (5), *Staphylococcus species* (2), *Aeromonas hydrophila* (1), *Citrobacter koseri* (1), *Escherichia coli* (1), *Raoultella + Streptococcus anginosus* (1)
**Pneumonia (13)**	*Streptococcus species* (7), *Pneumococcus* (4), *Mycoplasma* (1), *Staphylococcus intermedius* (1)
**Pyelonephritis (29)**	*Escherichia coli* (27), *Klebsiella oxytoca* (1), *Staphylococcus saprophyticus* (1)
**Gastroenteritis (12)**	*Campylobacter* (6), *Clostridium difficile* (4), *Salmonella + Campylobacter* (2)
**Erysipelas or cellulitis (8)**	*Streptococcus* (3), *Streptococcus pyogenes* (2), *Staphylococcus aureus* (1), *Enterococcus faecalis+Acinetobacter+ Bacteroides* (1), *Streptococcus pyogenes+Staphylococcus aureus+Enterobacter cloacae* (1)
**UTI, cystitis (5)**	*Escherichia coli* (3), *Escherichia coli+Klebsiella* (1), *Streptococcus intermedius+ Fusobacterium* (1)
**Pharyngitis (3)**	*Streptococcus pyogenes* (2), *Streptococcus* species (1)
**Abscess (2)**	*Escherichia coli+Enterococcus faecalis+Bacteroides vulgatus* (1), *Staphylococcus aureus* (1)
**Peritonitis (2)**	*Staphylococcus aureus* (1), *Streptococcus mitis* (1)
**Pericarditis (1)**	*Streptococcus constellatus* (1)

Supplementary Table 2. Detected viral pathogens causing the confirmed infectious diseases.

Diagnosis—(no.)	Pathogen(s)
**Upper respiratory tract infection (167)**	Influenza A virus (100), Influenza B virus (38), Respiratory syncytial virus (24), Rhinovirus (2), Adenovirus (1), Coronavirus (1), Metapneumovirus (1)
**Lower respiratory tract infection (102)**	Influenza A virus (52), Influenza B virus (26), Respiratory syncytial virus (18), Metapneumovirus (2), Human parainfluenza viruses (2), Epstein–Barr virus (1), Adenovirus (1)
**Epidemic nephropathy (10)**	Puumala orthohantavirus (10)
**Infectious mononucleosis (6)**	Cytomegalovirus (3), Epstein–Barr virus (3)
**Chickenpox (2)**	Varizella zoster virus (2)
**Dengue fever (1)**	Dengue virus (1)
**Encephalitis (1)**	Tick-borne encephalitis virus (1)
**Herpes (1)**	Herpes simplex virus (1)
**Gastroenteritis (1)**	Norovirus (1)

Supplementary Table 3. Group-specific antimicrobial prescriptions. In addition, antivirals Oseltamivir and Aciclovir were prescriped for 200 and 6 patients, respectively.

			DETECTED BACTERIA n = 193		DETECTED VIRUS n = 291		CLINICAL BACTERIA n = 83		CLINICAL VIRUS n = 20		FEVER WITH UNCLEAR REASON n = 254		BACTEREMIA n = 118		GRAM-positive BACTEREMIA n = 61		GRAM-negative BACTEREMIA n = 57		BACTERIAL PNEUMONIA n = 39		CLINICAL BACTERIAL PNEUMONIA n = 32		VIRUS PNEUMONIA n = 102		OTHER VIRUS INFECTION n = 189		PNEUMONIA WITH UNCLEAR REASON n = 95	
**Prescribed antibiotic**	**n**	**%**	**n**	**%**	**n**	**%**	**n**	**%**	**n**	**%**	**n**	**%**	**n**	**%**	**n**	**%**	**n**	**%**	**n**	**%**	**n**	**%**	**n**	**%**	**n**	**%**	**n**	**%**
**1** Cefuroxime	**453**	60	132	61	127	61	62	70	4	40	128	54	86	65	41	58	45	73	36	80	28	80	70	74	57	50	72	67
**2** Piperacillin / Tazobactam	**59**	7.8	19	8.7	20	9.7	3	3.4			17	7.2	10	7.5	4	5.6	6	9.7	1	2.2	1	2.9	8	8.5	12	10.6	6	5.6
**3** Ceftriaxone	**58**	7.6	20	9.2	18	8.7	5	5.6	5	50	10	4.3	11	8.3	6	8.5	5	8.1	1	2.2	1	2.9	5	5.3	13	11.5	3	2.8
**4** Levofloxacin	**29**	3.8	8	3.7	7	3.4	3	3.4			11	4.7	6	4.5	4	5.6	2	3.2	3	6.7	2	5.7	3	3.2	4	3.5	5	4.6
**5** Ciprofloxacin	**20**	2.6	8	3.7	1	0.5	2	2.2			9	3.8	2	1.5		0.0	2	3.2							1	0.9		
**6** Meropenem	**20**	2.6	5	2.3			5	5.6	1	10	9	3.8	3	2.3	2	2.8	1	1.6			2	5.7					2	1.9
**7** Doxycycline	**19**	2.5	2	0.9	8	3.9					9	3.8							2	4.4			1	1.1	7	6.2	4	3.7
**8** Metronidazole	**16**	2.1	4	1.8	2	1.0	1	1.1			9	3.8													2	1.8	1	0.9
**9** Amoxicillin	**15**	2.0			7	3.4					8	3.4											2	2.1	5	4.4	7	6.5
**10** Amoxicillin/Clavulanic acid	**13**	1.7	1	0.5	5	2.4					7	3.0	1	0.8	1	1.4			1	2.2			1	1.1	4	3.5	2	1.9
**11** Clindamycin	**10**	1.3	4	1.8	1	0.5	3	3.4			2	0.9	4	3.0	3	4.2	1	1.6							1	0.9		
**12** Cloxacillin	**6**	0.8	5	2.3	1	0.5							5	3.8	5	7.0									1	0.9		
**13** Moxifloxacin	**6**	0.8			3	1.4					3	1.3											2	2.1	1	0.9	3	2.8
**14** Azithromycin	**5**	0.7	1	0.5			1	1.1			3	1.3									1	2.9					2	1.9
**15** Cefalexin	**4**	0.5					1	1.1			3	1.3																
**16** Phenoxymethylpenicillin	**4**	0.5	1	0.5	2	1.0					1	0.4													2	1.8	1	0.9
**17** Vancomycin	**3**	0.4	2	0.9							1	0.4	2	1.5	2	2.8												
**18** Benzylpenicillin	**2**	0.3	1	0.5			1	1.1					1	0.8	1	1.4												
**19** Penicillin	**2**	0.3	1	0.5	1	0.5							1	0.8	1	1.4							1	1.1				
**20** Pivmecillinam	**2**	0.3									2	0.9																
**21** Tobramycin	**2**	0.3	1	0.5			1	1.1																				
**22** Cefalotin	**1**	0.1	1	0.5																								
**23** Ceftazidime	**1**	0.1	1	0.5									1	0.8	1	1.4												
**24** Clarithromycin	**1**	0.1									1	0.4																
**25** Fluconazole	**1**	0.1									1	0.4																
**26** Nitrofurantoin	**1**	0.1			1	0.5																	1	1.1				
**27** Norfloxacin	**1**	0.1			1	0.5																			1	0.9		
**28** Procaine benzylpenicillin	**1**	0.1					1	1.1																				
**29** Rifampicin	**1**	0.1			1	0.5																			1	0.9		
**30** Roxithromycin	**1**	0.1	1	0.5															1	2.2								
**31** Tigecycline	**1**	0.1									1	0.4																
**32** Trimethoprim	**1**	0.1			1	0.5																			1	0.9		
**SUM**	**759**	**100**	**218**	**100**	**207**	**100**	**89**	**100**	**10**	**100**	**235**	**100**	133	100	71	100	62	100	45	100	35	100	94	100	113	100	108	100
**Ab/patient**	**0.90**		**1.13**		**0.71**		**1.05**		**0.50**		**0.92**		1.11		1.13		1.09		1.25		1.03		0.90		0.61		1.08	

Supplementary Table 4. Demographic and clinical characteristics of the patients at baseline. COPD: Chronic obstructive pulmonary disease, DM1: Diabetes mellitus type 1, DM2: Diabetes mellitus type 2. ‡Independent-samples Kruskal-Wallis test, †Crosstab Chi-square tests.

Characteristic	Confirmed bacterial infection (N=193)	Confirmed viral infection (N=291)	Clinical bacterial infection (N=82)	Clinical viral infection (N=21)	Fever of unclear etiology (N=254)	p-value
Mean±SD age—yr‡	60.5±19.1	67.2±18.8	57.0±18.7	49.7±21.6	58.6±20.2	<0.001
Female sex—no. (%)†	118 (61.1)	151 (51.9)	36 (43.9)	15 (71.0)	119 (46.9)	0.003
Coexisting condition—no. (%)†						
Diabetes, DM1 or DM2	25 (13.0)	44 (14.4)	10 (12.2)	2 (9.5)	25 (9.8)	0.463
COPD	3 (1.6)	31 (10.7)	1 (1.2)	0 (0)	12 (4.7)	<0.001
Cancer	9 (4.7)	21 (7.2)	3 (3.7)	0 (0)	23 (9.1)	0.172
Inflammatory disease	20 (10.4)	46 (16.2)	8 (9.8)	2 (9.5)	70 (28.0)	<0.001
Pneumonia—no. (%)†	39 (20.2)	102 (35.1)	32 (39.0)	0 (0)	95 (37.4)	<0.001
Laboratory results—mean±SD‡						
Leukocytes—$\times 10^{-9}$/L	12.1±6.0	8.5±11.2	12.2±5.5	6.1±2.7	10.6±4.9	<0.001
CRP—mg/L	167±95.1	69.7±69.0	188±96.7		105±74.5	<0.001
PCT—ng/L	8291±18223	2164±6937	5175±13407	1330±4033	1454±4702	<0.001
FCBI-index	1659±5957	75.3±563	2069±14967	4.9±7.7	247±1260	<0.001

Supplementary Table 5. Characteristics of the monoclonal fluorescent-labelled antibodies used in the study.

Monoclonal Ab (mAb)	CD-marker	Label	Excitation*	Emission
**anti-Siglec9[1]**	CD329	FITC	**488nm (blue laser)**	**525nm bandpass filter, green fluorescence**
**anti-human CR1[2]**	CD35	PE	**488nm (blue laser)**	**575nm bandpass filter, orange fluorescence**
**anti-human FcγRI[3]**	CD64	PerCP-Vio700	**488nm (blue laser)**	**665nm longpass filter, red/infrared fluorescence**
**anti-MHC class I molecule[4]**	-	AF647	**638nm (red laser)**	**675nm bandpass filter, red fluorescence**

1. anti-human CD329 (Clone 191240)) was purchased from R&D systems
2. anti-human CD35 (Clone E11) was purchased from Ancell
3. anti-human CD64 (Clone 10.1.1) was purchased from Miltenyi Biotec
4. anti-human MHC class I molecule (Clone W6/32) was purchased from R&D systems

Supplementary Table 6. Reproducibility test number 1. The influence of different gate settings during flow cytometric data analysis on final FCBI-index values. Ten consecutive FCBI-index calculations were performed starting with fresh gate settings every time using the flow cytometric raw data of six patients as a test material. SD%: proportion of standard deviation (SD) from mean value.

	**FCBI-index values**					
	**patient 1**	**patient 2**	**patient 3**	**patient 4**	**patient 5**	**patient 6**
gate setting 1	7336	867	418	5.075	0.433	0.0085
gate setting 2	7849	971	420	5.208	0.420	0.0090
gate setting 3	7078	911	440	5.211	0.439	0.0086
gate setting 4	6831	915	410	5.536	0.424	0.0087
gate setting 5	7057	902	437	5.474	0.443	0.0087
gate setting 6	7368	907	431	5.486	0.427	0.0085
gate setting 7	7191	862	394	5.234	0.420	0.0085
gate setting 8	6833	852	431	5.444	0.424	0.0084
gate setting 9	7218	877	408	5.505	0.423	0.0086
gate setting 10	7529	852	432	5.490	0.418	0.0085
mean	*7229*	*892*	*422*	*5.366*	*0.427*	*0.0086*
SD	*296*	*35*	*14*	*0.157*	*0.008*	*0.0002*
SD%	***4.09***	***3.95***	***3.36***	***2.93***	***1.88***	***1.85***

Supplementary Table 7. Reproducibility test number 2. The influence of sample handling protocol (Incubation of the whole blood sample with the receptor-specific antibodies, red blood cell lysis, and flow cytometry run) on final FCBI-index values was tested by repeating the sample handling protocol simultaneously in five separate test tubes. FCBI-index values of five separate measurements were determined using constant gate settings. SD%: proportion of standard deviation (SD) from mean value.

	**FCBI-index value**
	**patient 7**
tube 1	3.30
tube 2	3.31
tube 3	3.29
tube 4	3.63
tube 5	3.46
mean	*3.41*
SD	*0.16*
SD%	***4.58***

Supplementary Table 8. Complete matrix presenting significant p-values of pairwise comparisons between different respiratory tract infection subgroups for Fig. 7 data. Probability values for FCBI-index, CDRP and PCT data are highlighted in black, magenta and green, respectively.

SAMPLE 2 \ SAMPLE 1		Pneumonia with unknown etiology (n=95)	Confirmed pneumonic InfA (n=52)	Confirmed pneumonic InfB (n=26)	Confirmed pneumonic RSV (n=18)	Confirmed NON-pneumonic InfA (n=100)	Confirmed NON-pneumonic InfB (n=38)	Confirmed NON-pneumonic RSV (n=24)
Bacterial pneumonia (confirmed or clinically diagnosed) (n=71)	FCBI-index	0.014	<0.001	<0.001	0.012	<0.001	<0.001	<0.001
	CRP	0.001	<0.001	0.001	<0.001	<0.001	<0.001	<0.001
	PCT	<0.001	0.017	-	<0.001	<0.001	<0.001	<0.001
Pneumonia with unknown etiology (n=95)	FCBI-index		<0.001	-	-	<0.001	<0.001	<0.001
	CRP		0.001	-	-	<0.001	<0.001	<0.001
	PCT			0.012	-	-	-	-
Confirmed pneumonic InfA (n=52)	FCBI-index			-	-	-	-	-
	CRP			-	-	-	-	-
	PCT			-	-	-	-	0.035
Confirmed pneumonic InfB (n=26)	FCBI-index				-	0.029	0.012	-
	CRP				-	0.01	0.002	-
	PCT				0.003	<0.001	<0.001	<0.001
Confirmed pneumonic RSV (n=18)	FCBI-index					0.006	0.002	-
	CRP					-	-	-
	PCT					-	-	-
Confirmed NON-pneumonic InfA (n=100)	FCBI-index						-	-
	CRP						-	-
	PCT						-	-
Confirmed NON-pneumonic InfB (n=38)	FCBI-index							-
	CRP							-
	PCT							-
